# Brain Network Dynamics of Local and Global Predictive Processing in Aging

**DOI:** 10.1002/advs.77857

**Published:** 2026-09-27

**Authors:** Mathias Houe Andersen, Gemma Fernández‐Rubio, David R. Quiroga‐Martinez, Mattia Rosso, Mathias Klarlund, Kit Melissa Larsen, Hartwig Roman Siebner, Morten L. Kringelbach, Peter Vuust, Leonardo Bonetti

**Affiliations:** ^1^ Center For Music in the Brain Department of Clinical Medicine Aarhus University & The Royal Academy of Music Aarhus/Aalborg Denmark; ^2^ Danish Research Centre For Magnetic Resonance Department of Radiology and Nuclear Medicine Copenhagen University Hospital – Amager and Hvidovre Hvidovre Denmark; ^3^ Faculty of Health and Medical Sciences University of Copenhagen Copenhagen Denmark; ^4^ Department of Psychology University of Copenhagen Copenhagen Denmark; ^5^ IPEM Institute for Systematic Musicology Ghent University Ghent Belgium; ^6^ Department of Neurology Copenhagen University Hospital Bispebjerg and Frederiksberg Copenhagen Denmark; ^7^ Department of Psychiatry University of Oxford Oxford UK; ^8^ Centre For Eudaimonia and Human Flourishing Linacre College University of Oxford Oxford UK

**Keywords:** aging, brain networks, phase space, predictive coding, principal component analysis (PCA), recurrence quantification analysis (RQA)

## Abstract

Cognitive aging is widely associated with a progressive weakening of neural responses associated with predictive brain mechanisms. This view is supported by decades of electrophysiological studies reporting attenuated mismatch responses in older adults. Yet the literature remains inconsistent, suggesting that aging may not uniformly attenuate such responses. One possibility is that aging exerts differential effects depending on task demands. Here we aim to separate whole‐brain networks underlying deviance processing in source‐reconstructed magnetoencephalography (MEG) data from 37 younger and 40 older adults performing the auditory local–global paradigm. Network decomposition revealed three temporally overlapping subsystems. Aging exerted selective effects across these networks. Early sensory deviance responses were enhanced within a network recruiting auditory cortices and medial cingulate regions, whereas later cognitive processes were attenuated in older adults. The level of multivariate recurrence across these networks was preserved with aging, while the processing of sensory violations induced more recurrence and less divergence relative to pattern‐based violations in both groups. This age‐related increase in sensory‐related mismatch responses challenges the prevailing view that sensory deviance processing simply declines with age. These results suggest that in aging, neural responses may be differentially distributed across distinct neural systems, amplifying sensory‐based processes while weakening cognitively demanding mechanisms.

## Graphical abstract

Separation of concurrent whole‐brain networks in source‐reconstructed magnetoencephalography (MEG) data suggests that healthy aging reorganizes, rather than uniformly attenuates, neural responses elicited from hierarchical auditory violations. Enhanced early sensory deviance processing alongside reduced higher‐order cognitive responses suggests a large‐scale reorganization of the neural responses associated with predictive processing in the aging brain.

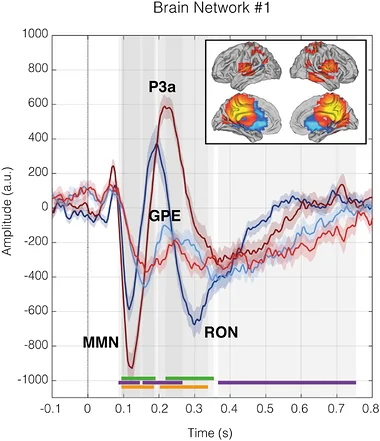

## 1 Introduction

Predictive coding posits that perception arises from the brain's continuous attempts to anticipate incoming sensory signals and that internal models are updated when sensory evidence violates those expectations. Such mismatches generate neural responses associated with prediction error signaling at multiple hierarchical and temporal scales within the nervous system [1, 2, 3, 4, 5, 6]. In the auditory domain, predictive processing spans a broad range, from local predictions about immediate tone features to global expectations about abstract, higher‐order regularities unfolding over extended time windows [7, 8, 9].

Age‐related changes in neural responses associated with predictive processing have been studied most extensively at the local sensory level. The prevailing view is that aging weakens prediction error signaling, particularly for simple sensory violations. This interpretation is supported by numerous electrophysiological studies reporting reduced mismatch responses in older adults and has often been framed as an age‐related shift in the balance between top‐down priors derived from internal models and bottom‐up evidence sampled through sensory input channels [10, 11, 12, 13, 14, 15, 16, 17, 18]. As sensory systems deteriorate with age, incoming signals may become less precise, encouraging the brain to rely more heavily on prior expectations and less on noisy incoming sensory input [19, 20]. In such a framework, attenuated prediction error signaling would be an adaptive consequence of reduced sensory reliability [21].

Yet this account remains incomplete. Findings across the aging literature are not fully consistent with this account, and the evidence for a uniform decline in sensory prediction error signaling, indexed by the mismatch negativity (MMN) and P3a response, is weaker than often assumed. Although many studies report reduced MMN responses in older adults [17, 22, 23, 24], meta‐analytic work also finds that a substantial number of experiments detect no age‐related MMN reduction [16]. In several cases, preserved mismatch responses have been attributed to altered recruitment of attention‐related neural processes [15, 25, 26, 27, 28, 29, 30]. Similarly, in the relatively few studies using paradigms that probe both local sensory violations and higher‐order pattern violations, age effects have not been uniform across levels. While MMN appears unchanged with age, cognitive responses previously associated with the processing of contextual pattern violations (global prediction error, GPE) and late cognitive responses associated with reorienting neural resources toward the task at hand (reorienting negativity, RON) appear more consistently attenuated with age [7, 8, 21, 31, 32, 33]. This pattern raises the possibility that aging does not uniformly reduce neural responses associated with predictive processing but rather alters how distinct subsystems are engaged.

A major limitation of prior work is that most studies have relied on scalp‐level magneto‐ or electroencephalography (M/EEG) analyses [34, 35] and on a component‐centric logic focused on canonical event‐related responses such as MMN, P3a, the GPE response, and RON [36, 37, 38, 39, 40]. These responses are useful markers, but they do not directly reveal the large‐scale brain networks that generate them, nor how such networks may differ across age. Source‐localization studies suggest that local violations recruit bilateral fronto‐temporo‐parietal regions, including Heschl's gyrus and surrounding auditory cortex [7, 41], whereas global violations engage broader fronto‐parieto‐temporal systems overlapping with global workspace accounts of conscious access [7, 42, 43, 44]. However, these network‐level systems have only rarely been disentangled in relation to specific predictive responses and have not been compared across age in order to characterize their temporal dynamics.

To address this gap, we apply a recently developed method, BROADband Network Estimation via Source Separation (BROAD‐NESS) [45, 46], to separate simultaneous, orthogonal brain networks from source‐reconstructed MEG data. This approach makes it possible to move beyond single sensor‐level components and identify concurrent large‐scale networks underlying neural responses associated with predictive processing. Importantly, BROAD‐NESS also enables a dynamical characterization of these networks. By embedding the dominant component time series into a low‐dimensional phase space, brain activity can be described as trajectories through a shared network state space. Recurrence quantification analysis (RQA) applied to these trajectories provides quantitative indices of stability, predictability, persistence, and complexity, revealing how often the system revisits similar network states over time [47, 48, 49, 50]. This allows us to ask not only which networks support neural responses associated with prediction error signaling, but also whether the dynamical regime of these processes differs between sensory and pattern violations, and whether these distinctions are preserved in aging.

In the present study, we applied BROAD‐NESS to source‐reconstructed MEG data from younger and older adults performing the auditory local–global paradigm. Our aims were threefold: (i) to identify the dominant whole‐brain networks involved in local and global deviance processing; (ii) to reconstruct group‐level network time series in order to relate these networks to canonical event‐related field (ERF) responses; and (iii) to quantify how the phase‐space dynamics and recurrence structures of these networks differ between sensory and pattern violations and across age. By doing so, we test the conventional uniform decline account, which posits that ERF responses associated with predictive processes attenuate with age independent of task demands and characterize the network configurations underlying these processes.

## 2 Results

### 2.1 Overview of the Experimental Design and MEG Source Reconstruction

**FIGURE 1:**
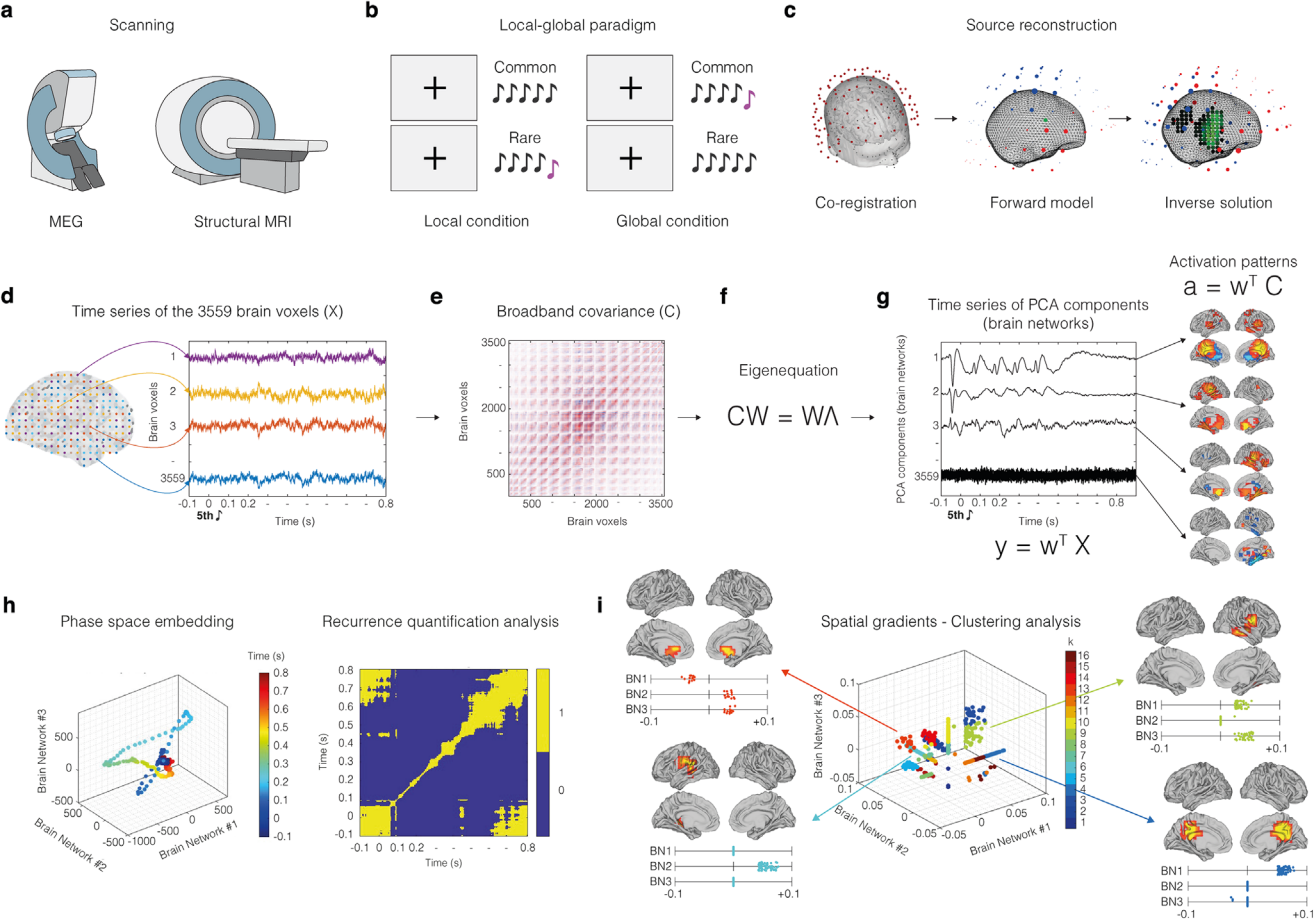
Methodology – BROADband network estimation via source separation (BROAD‐NESS). (a) MEG was used to collect neurophysiological data while participants listened to the auditory local‐global paradigm. (b) Auditory sequences of five tones, containing either five repetitive tones or four repetitive tones and a deviating fifth tone, were presented in random order. Global deviations were achieved by switching the probability of occurrences between the two sequences, producing a pattern violation. (c) Co‐registration was performed between MEG data and anatomical MRI scans of each individual. To reconstruct the neural sources that generated the signal recorded by the MEG, a single shell forward model was used. The inverse solution was estimated through beamforming. (d) Source reconstruction yielded time series data for 3559 brain voxels from an 8 mm grid brain parcellation at a 250 Hz sampling frequency. (e) The covariance matrix (C) was computed from the broadband voxel data matrix. (f) Principal Component Analysis (PCA) was computed by solving the eigenequation CW = WΛ for the eigenvectors (W) to find the weighted combinations of brain voxels into orthogonal components, which reflected brain networks; associated eigenvalues (Λ) express the amount of variance explained by each network component. (g) Network activation time series (y) were derived by applying the spatial filters (w) to the voxel data matrix (X). The same filters were applied to the covariance matrix (C) to compute the spatial activation patterns (a), representing the projection of components in voxel space. (h) Temporal embedding and recurrence quantification analysis were applied to the network time series to investigate their dynamic temporal properties. Phase space plots (left) and thresholded recurrence plots (right) captured differences in trajectory recurrence across experimental conditions. (i) To examine the spatial organization of voxel contributions to each brain network, we performed a spatial gradient embedding and clustering analysis based on the spatial activation patterns of the three main brain networks. An unsupervised clustering procedure applied to this space revealed anatomically interpretable voxel groups, including clusters of voxels selectively involved in one or two networks.

A total of 77 participants (37 younger adults, 40 older adults) engaged in an adapted version of the auditory local‐global paradigm [7] during magnetoencephalography (MEG) recordings (Figure 1a,b). The paradigm consisted of 160 trials per block for a total of 4 blocks. Each block included a combination of patterns consisting of either five repetitive tones or patterns where the fifth tone deviated in pitch (Figure S1). The local‐global paradigm was designed to elicit local neural responses from sensory violations at the single‐tone level and global neural responses from violations at the pattern level across blocks. The paradigm shifted between two block types, where the number of occurrences in which the fifth tone differed in pitch was changed to create a global violation of the expected pattern. In both block types, a target tone requiring a button press was also presented to ensure continued attention toward the paradigm.

After performing pre‐processing of the MEG data (see Methods for details), we computed source reconstruction and obtained the difference wave by subtracting responses to standard stimuli from responses to deviant stimuli. We employed a single‐shell forward model alongside a beamforming approach as the inverse solution, using an 8 mm grid that corresponded to 3559 brain voxels (Figure 1c,d). This procedure produced a time series for each reconstructed brain voxel, returning a matrix of 3559 brain voxels × 326 time points, independently for each participant and experimental condition.

### 2.2 Deriving Brain Networks via PCA

PCA was conducted on the reconstructed time series of the 3559 brain voxels, averaged across participants and conditions, to identify simultaneously operating broadband brain networks (Figure 1e–g). Effective dimensionality (ED) [51] was computed from the PCA eigenvalue spectrum to quantify the number of components that meaningfully contribute to the data (see Methods for details). This procedure identified three significant PCs (ED = 2.835), which accounted for 51.78%, 24.57%, and 15.12% of the variance in the data. Each component was interpreted as a distinct brain network based on its spatial activation pattern, which was obtained by multiplying the eigenvector of the component with the covariance matrix of the data.

**FIGURE 2:**
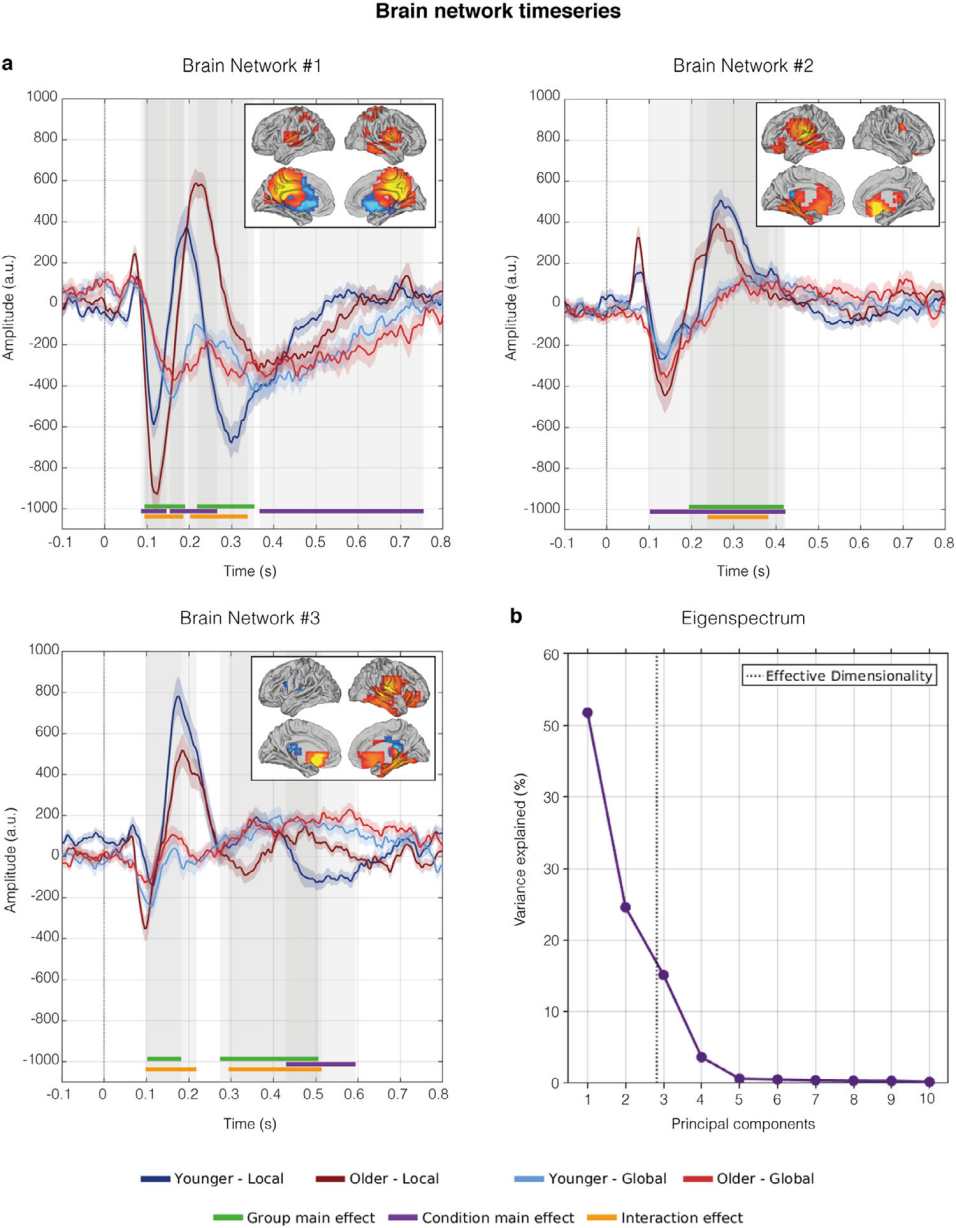
Time series and spatial activation patterns of the main brain networks. The figure illustrates the time series and spatial activation patterns of the three brain networks that explained the highest variance (51.78%, 24.57%, and 15.12%, respectively). These networks were estimated using PCA within the BROAD‐NESS framework, computed on the data averaged across conditions and participants. (a) Independent time series for each participant, brain network, and experimental condition were generated using PCA‐derived weights from the averaged data. The individual time series were then averaged across participants, as shown in the plots. Shaded areas represent standard errors of the mean. The brain templates illustrate the spatial extent of the networks, with yellow voxels contributing the most and light blue voxels contributing the least to the time series. Only voxels with values exceeding the mean by more than one standard deviation in absolute terms are depicted. Significant group, condition, and interaction effects are indicated by green, purple, and yellow lines. Time = 0 s. indicates the onset of the fifth tone in the local‐global paradigm. The weights of the spatial activation patterns were normalized independently for each PC. b) The eigenspectrum for the first 10 principal components and effective dimensionality (ED = 2.835) indicate the relevance of the first three principal components (dashed line).

As illustrated in Figure 2, the first identified brain network comprised bilateral auditory cortices such as Heschl's gyri, Rolandic Operculum, the Medial and Posterior Cingulate Cortices, the Precuneus and the Insula. The second network involved the auditory cortex, including Heschl's gyrus and the Fusiform gyrus, the Hippocampus and Rolandic Operculum of the left hemisphere, and bilateral frontal areas such as Rectus, Olfactory Bulb, and Subgenual Anterior Cingulate Cortices (ACC). The third network showed a closely mirrored pattern of the second network, displaying connections between Heschl's gyrus, Fusiform gyrus, Hippocampus, Rolandic Operculum, Caudate, and Supramarginal gyrus of the right hemisphere and bilateral frontal areas including Rectus, Olfactory Bulb, and Subgenual ACC. For specific MNI coordinates and voxel counts within each brain area, see Tables S1 and S2. The remaining networks were deemed less relevant as they explained considerably less variance and fell below the effective dimensionality (ED) threshold.

After deriving the time series in each network at participant level, statistical analyses examining age group, condition, and interaction effects were conducted for each of the first three brain networks. This analysis revealed several time windows of significant differences between the local and global conditions, between age groups, and between the interactions of condition and age group (Figure 2). Regarding age group effects, a general pattern was observed, where early predominantly sensory‐based responses were increased, while late and more cognitive responses were attenuated in older adults relative to younger adults. Specifically, in Brain Network #1, older adults displayed enhanced local MMN and P3a responses, and an attenuated RON response compared to younger adults. Furthermore, the GPE response was reduced in older adults. In Brain Network #2 and #3, younger adults displayed an increased P3a response, while the RON response was also increased among younger adults in Brain Network #3. Several significant condition and interaction differences were found throughout the time window. Detailed cluster onsets/offsets and cluster‐level *p*‐values are summarized in Table S3. See Figure S2 for details regarding the detection of neural responses.

In addition, we performed three robustness checks to investigate whether these main results of the ERF responses were also observed via i) traditional scalp analyses (Figure S7 and Table S12), ii) when behavioral outliers were removed (Figures S8 and S9; Table S13), and iii) when trials were randomly permuted and split into two halves (Figure S10.1 and S10.2; Tables S14.1 and S14.2). These tests displayed the same pattern of ERF response deflections, where early sensory‐based responses (local MMN, P3a) were observed to be enhanced with aging, while late and cognitive responses (RON, GPE) were observed to be attenuated in older adults.

Furthermore, by observing the mean latency of key ERF responses across these three main brain networks in each group for each condition, a specific order of elicited responses was indicated in both the local and the global conditions (Figure S2 and Table S4.1). To evaluate this further, we tested for significant latency differences in each ERF component between the three main networks (Tables S4.2–S4.4). We observed a statistically significant ordering across the three networks for the local MMN response (*p* = 4.50 × 10^−6^), the local P3a response (*p* = 2.44 × 10^−14^), and the global MMN response (*p* = 9.08 × 10^−13^). For the local MMN, the latency order was PC #3 → PC #1 → PC #2 (mean latencies: 102.6, 118.4, and 130.1 ms, respectively), and all pairwise comparisons were significant after FDR correction (all *p*FDR < 0.037). A similar ordering was observed for the local P3a response, with latencies following the sequence PC #3 → PC #1 → PC #2 (mean latencies: 180.4, 190.2, and 278.1 ms, respectively). Pairwise comparisons showed significantly later responses in PC #2 relative to both PC #1 and PC #3 (both *p*FDR < 5.3 × 10^−^^8^), whereas no significant latency difference was observed between PC #1 and PC #3 (*p*FDR = 0.080). For the global MMN, the latency order was PC #3 → PC #2 → PC #1 (mean latencies: 104.8, 140.7, and 162.2 ms, respectively), with all pairwise comparisons remaining significant after FDR correction (all *p*FDR < 0.002).

These findings indicate that the sensory‐based MMN‐related activity was consistently initiated in Brain Network #3, corresponding predominantly to the right auditory cortex. During local deviance processing, activity was subsequently elicited in Brain Network #1 rooted in a central cingulate network before Brain Network #2 was activated, which is situated predominantly in the left auditory cortex. The same PC #3 → PC #1 → PC #2 temporal sequence was also observed during local P3a processing, suggesting that this network organization extends beyond early mismatch detection into later stages of deviance evaluation. In contrast, during global deviance processing, ERF responses were initiated in Brain Network #3, followed by Brain Network #2 and Brain Network #1. This indicates a more widespread activation pattern, where the right hemisphere was first activated, followed by the left hemisphere and terminating in a central cingulate network.

### 2.3 Phase Space and Recurrence Quantification Analysis

To further characterize the temporal organization of BROAD‐NESS‐derived brain network dynamics, we embedded the three dominant networks into a joint phase space and quantified their recurrence structure [45]. A cluster‐based permutation test on the Euclidean distance between the 3D mean phase‐space trajectories (5,000 permutations; cluster‐level family‐wise error correction) was used to identify time periods during which trajectories differed between the two age groups and between conditions across both age groups. Here, a greater excursion of the phase‐space trajectory indicates a greater Euclidean magnitude of the combined activation across the three concurrently expressed brain networks (PC #1, PC #2, PC #3), rather than a stronger activation of any single network. In this context of the phase space analysis, more pronounced means further away from the midpoint of the phase space, where (PC #1, PC #2, PC #3) is equal to (0, 0, 0).

**FIGURE 3:**
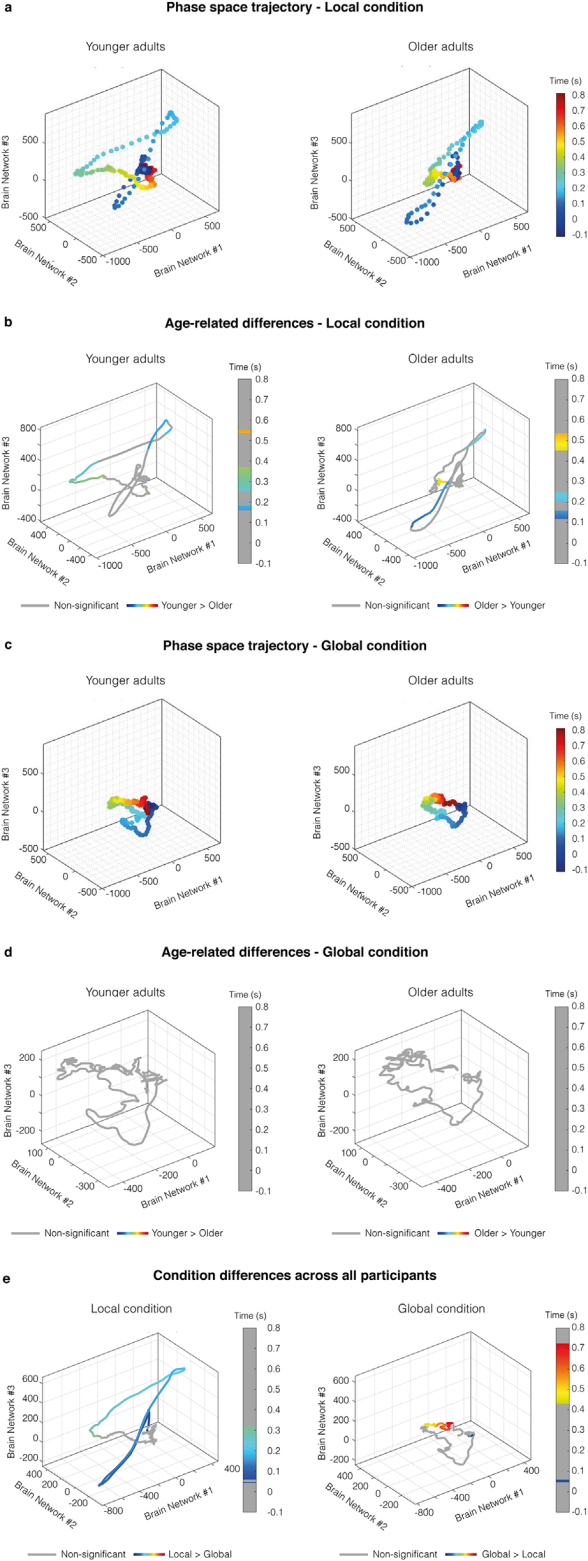
Phase space embedding of BROAD‐NESS‐derived brain network dynamics. This figure depicts phase space trajectories averaged across participants for each experimental condition and age group, obtained by embedding the time series of the three primary BROAD‐NESS networks into a 3D state space. Each dot represents the state of the brain at a given time point, color‐coded by time (in seconds after the onset of the fifth tone). For specific values at each time point, see Table S5. (a) Plots of the mean phase space trajectory in the local condition in each age group. (b) The mean phase space trajectory in the local condition within each age group, with statistically significant age group differences colored. (c) Plots of the mean phase space trajectory in the global condition in each age group. (d) The mean phase space trajectory in the global condition within each age group, with statistically significant age group differences colored. (e) Mean phase space trajectories in each condition across both age groups, with statistically significant condition differences colored.

In the local condition (Figure 3a,b), significant age group differences were observed during various time windows, as younger adults showed a more pronounced phase space trajectory from 160 to 180 ms, 252 to 372 ms, and 540 to 556 ms post‐stimulus. Conversely, older adults exhibited a pronounced phase space trajectory at 120–160 ms, 204–252 ms, and 456–540 ms post‐stimulus. The earliest interval of significant effects (120–180 ms) overlapped primarily with the local MMN responses across the three brain networks (Table S4.1) and was predominantly characterized by a greater excursion of phase‐space trajectories in older adults than younger adults. During the subsequent interval (204–372 ms), spanning the P3a and the RON response, older adults exhibited a pronounced phase‐space trajectory during the P3a response, while younger adults exhibited a pronounced trajectory during the RON response. Finally, during the late interval of significant differences (456–556 ms), corresponding to very late deflections following the RON response, a short interval was dominated by younger adults, while older adults predominantly exhibited a greater excursion in their phase‐space trajectory. These differences appear to be driven by a slower return to baseline in older adults in Brain Network #1. In contrast, no significant differences between younger and older adults were observed in the global condition (Figure 3c,d).

Across all participants, the local and global conditions differed significantly (Figure 3e). The local condition exhibited a more pronounced phase‐space trajectory compared to the global condition from 60 to 316 ms, encompassing the MMN, P3a, and RON responses across the three brain networks. Conversely, the global condition exhibited a greater excursion in the phase‐space trajectory from 432 to 724 ms, corresponding to the later stages of processing following the principal ERF components. A brief transition from 44 to 60 ms was statistically significant but occurred before the canonical deviance‐related ERF components.

Furthermore, in both the three and two dimensional phase space trajectories, a more distinct coactivation and deactivation of brain networks was observed in the local condition compared to the global condition. Similarly, when inspecting the phase space trajectories of two of the three brain networks against each other in all three possible combinations, younger adults exhibited a more sharply defined time‐specific coactivation and deactivation of singular brain networks compared to slower and less distinct transitions observed among older adults (Figure S3.1).

**FIGURE 4:**
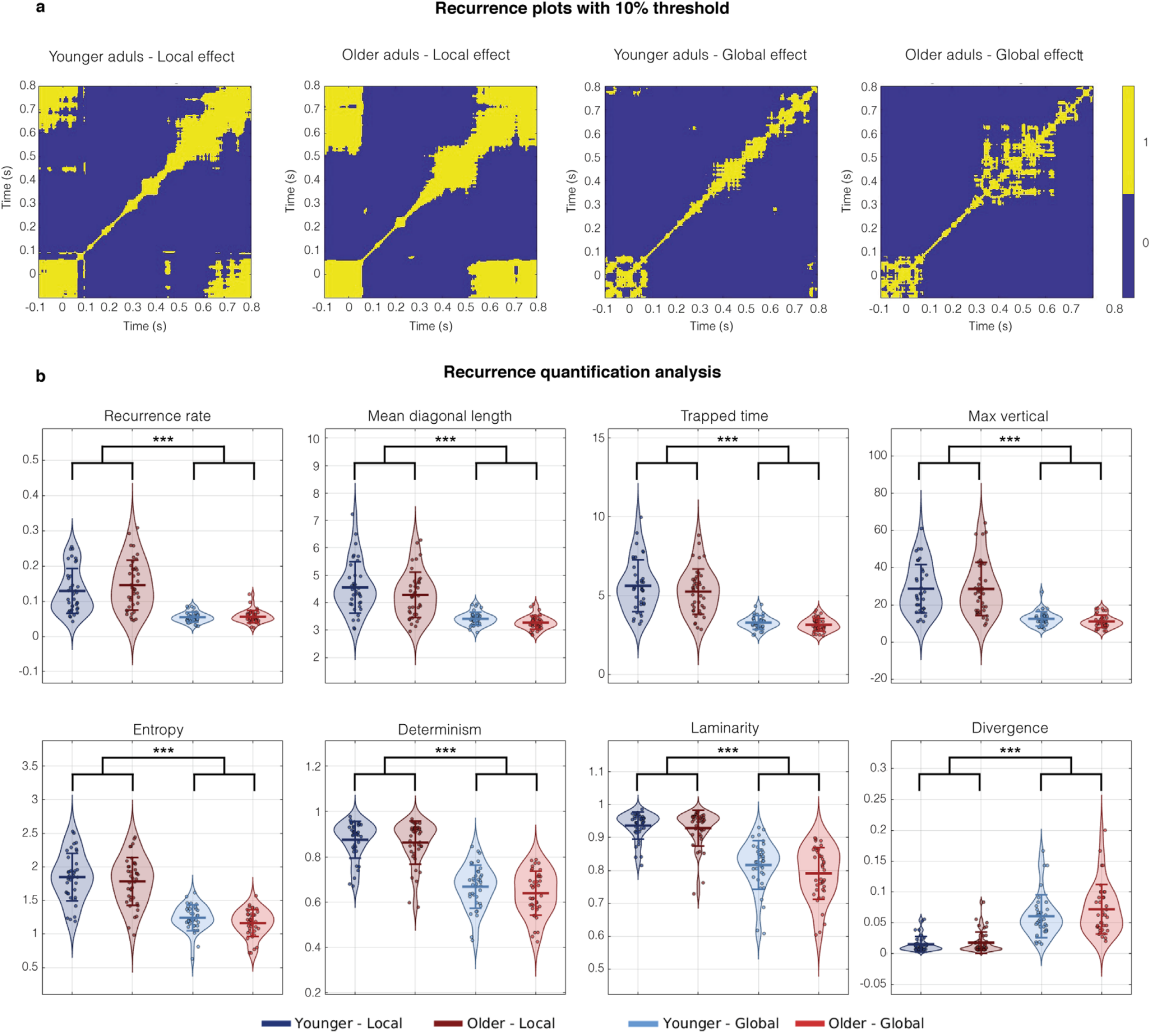
Recurrence quantification analysis of BROAD‐NESS‐derived brain network dynamics. (a) Recurrence plots computed from the phase space trajectories shown in Figure 3 are averaged across participants, depicting pairwise Euclidean distances between time points above and below a 10% threshold. Blue indicates larger distances (i.e., less similar brain states) that fall below the 10% threshold, while yellow indicates greater similarity and temporal recurrence above the 10% threshold. Figure S4 depicts the unthresholded distance matrices subserving these recurrence plots. (b) Violin plots of eight essential recurrence quantification analysis metrics. The violin plots display the smoothed distribution of the data, along with the individual values, the group means, and bars representing one standard deviation. The plots illustrate the statistically significant comparison between the local and global conditions. These metrics quantify the predictability, complexity, and temporal persistence of network state trajectories. Significance levels: * *p* < 0.05, ** *p* < 0.01, *** *p* < 0.001; all *p*‐values were significant after false discovery rate (FDR) correction.

When inspecting the recurrence plots derived from these trajectories (Figure 4a), there was a clear tendency for greater recurrence in the local condition across groups. Recurrence here refers to how often a previously occupied state is revisited, reflecting how often the system's dynamics return to similar configurations over time. For eight central recurrence metrics (such as recurrence rate, trapping time and entropy), effects of age group, condition and interaction were examined (Figure 4b). All metrics showed significant condition effects (all FDR‐corrected *p*‐values ≤ 1.6 × 10^−^^9^), with no significant main effects of group and no significant group × condition interactions (all FDR‐corrected *p*‐values ≥ 0.16). Specifically, all metrics except divergence were increased in the local compared to the global condition, whereas divergence was increased in the global condition. These results indicate that, relative to global pattern processing, the local deviance condition is associated with more recurrent, predictable, and temporally persistent network trajectories, while global processing is characterized by faster separation of trajectories, less stable, and more exploratory dynamics.

### 2.4 Spatial Gradient Embedding of BROAD‐NESS‐Derived Network Topographies

**FIGURE 5:**
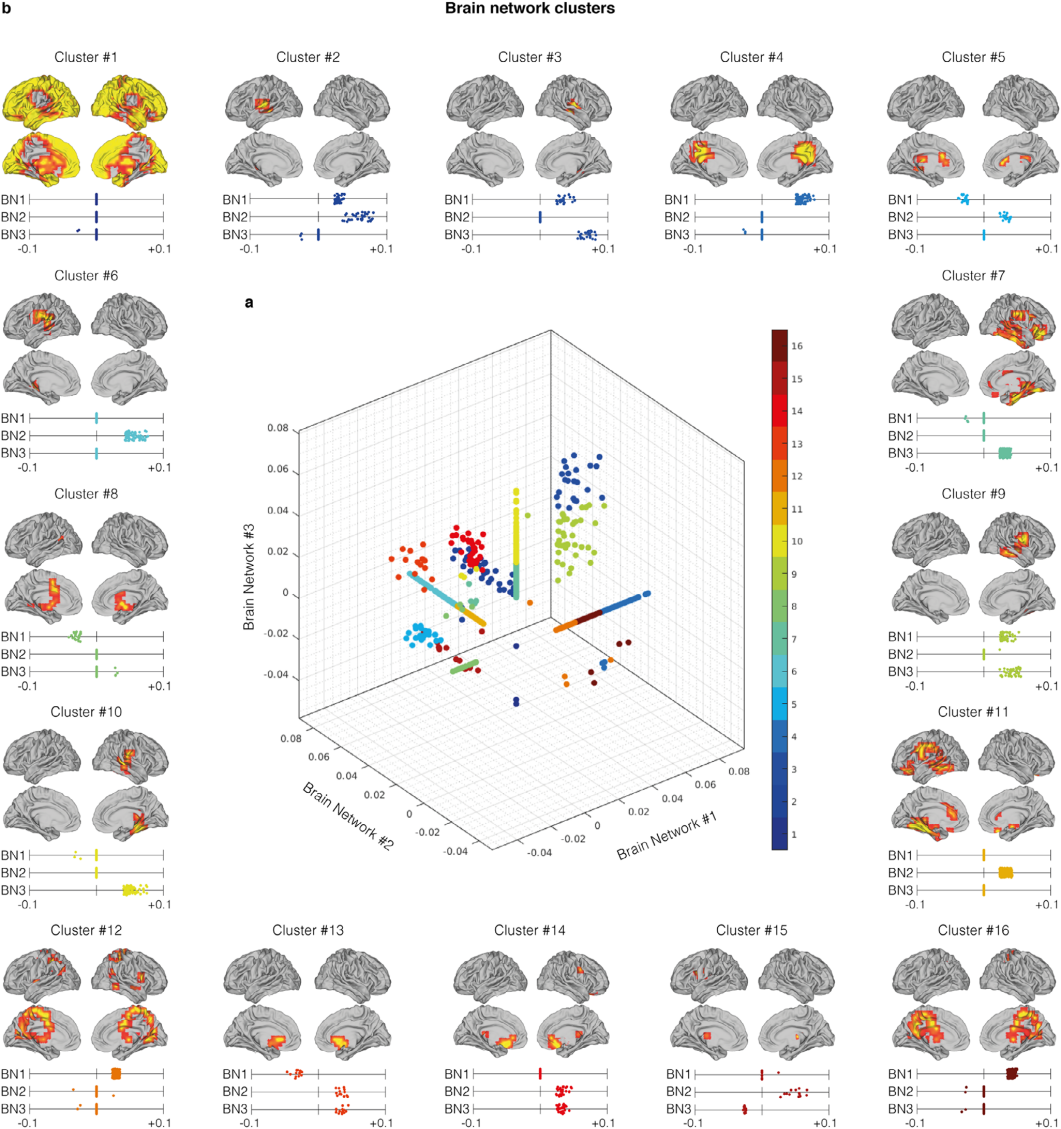
Spatial gradient embedding and voxel clustering analysis. This figure illustrates the analysis of voxel‐wise spatial activation patterns derived from the three main BROAD‐NESS brain networks. (a) 3D scatter plot showing the distribution of all brain voxels according to their spatial activation values in the brain networks obtained from the first three principal components revealing the underlying network involvement. The colored voxels reveal the spatial gradients of the sixteen clusters identified by the optimal clustering solution, highlighting distinct patterns of co‐involvement across the three networks. (b) Brain template projections of the sixteen clusters from the optimal solution and their corresponding activation coefficients on each of the three main brain networks. These maps illustrate the spatial distribution of voxel groups identified in the central scatter plot revealing coherent and anatomically interpretable clusters, including shared, selective, and opposing contributors to the three BROAD‐NESS networks. See Figure S6 for a histogram of silhouette coefficients across 5,000 clustering iterations for each tested solution (*k* = 2–40). The peak at *k* = 16 confirms the optimal number of spatial clusters, based on maximum silhouette coefficient consistency.

To further characterize the spatial organization of the BROAD‐NESS‐derived networks, we applied a clustering approach to the voxel‐wise activation maps of the first three principal components underlying the three main brain networks. Each voxel was embedded in a 3D space according to the contribution of the voxel to each brain network (thresholded at mean ± 1 standard deviation), enabling a compact spatial representation of network co‐involvement across the brain. Clustering analyses using k‐means (performed on z‐scored values and repeated 100 times per *k*, ranging from two to 40) revealed that a 16‐cluster solution provided the most robust and reproducible partitioning of voxels in this space, as evaluated using silhouette coefficients. To ensure the reliability of this clustering solution, we repeated the entire clustering and silhouette evaluation procedure 5,000 times and consistently found that *k* = 16 yielded the highest silhouette scores across most repetitions. This optimal solution is visualized in Figure 5, which shows the identified clusters projected back onto anatomical brain templates, while detailed information on the brain voxels forming these sixteen clusters is reported in Tables S6–S8. For full transparency, Figure S5 displays clustering results and silhouette coefficients across the full range of *k* values, highlighting the robustness of the solution and the consistency of the results across adjacent clustering solutions. The sixteen‐cluster structure provided a clear and interpretable mapping of how different voxel populations contributed to the three dominant networks. This analysis confirmed the presence of voxels strongly engaged in two of the three main brain networks, particularly within the auditory, cingulate and frontal cortices. Two examples are given: (i) Cluster #2 identified a group of voxels that contribute to the common auditory processes involved in Brain Network #1 and #2 within the left Heschl's gyrus, Rolandic Operculum and Insula. (ii) Cluster #14 isolated a group of voxels that exclusively contributed to the frontal regions involved in Brain Network #2 and #3 within bilateral Rectus, and the right Olfactory Bulb and Subgenual Anterior Cingulate Cortex. We also observed voxels selectively contributing to only one network. For instance, bilateral Precuneus and the Posterior and Medial Cingulate cortices in Brain Network #1 (e.g. Cluster #4, #12 and #16), the left auditory cortex including Heschl's gyrus in Brain Network #2 (Cluster #6 and #11) and the right auditory cortex including the Heschl's gyrus in Brain Network #3 (Cluster #9 and #10). Finally, the analysis revealed a spatially coherent cluster of voxels with minimal or no contribution to either network, likely representing non‐task‐specific background activity (Cluster #1). Voxels within this cluster covered large parts of the frontal and occipital lobe and medial regions minimally implicated in the local‐global paradigm.

### 2.5 Brain Network Modularity Analysis

To assess whether the large‐scale brain networks engaged by the local–global paradigm were more constrained or more broadly distributed, we applied BROAD‐NESS at the individual level and derived effective dimensionality from the eigenspectrum of the resulting covariance matrices. ED quantifies the effective number of orthogonal components contributing to the signal, with higher values indicating broader and less constrained network involvement.

**FIGURE 6:**
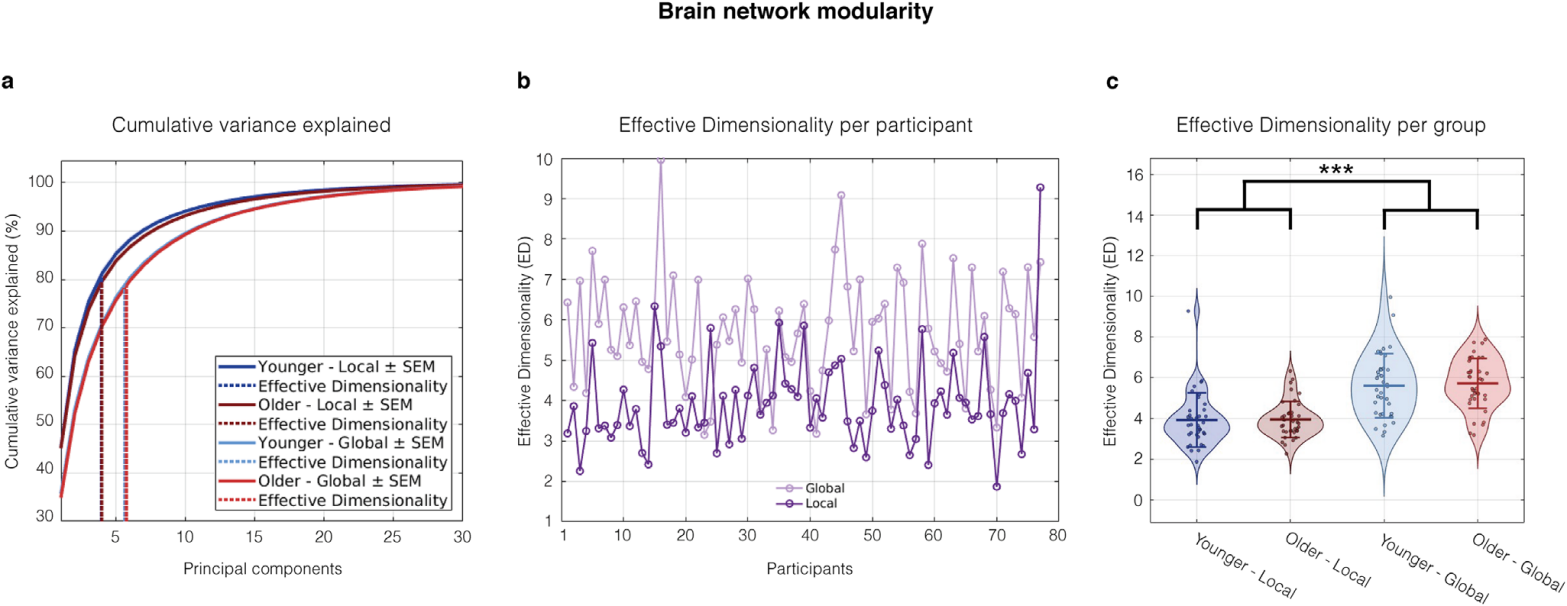
Brain network modularity analysis. (a) Cumulative variance explained by principal components when BROAD‐NESS is applied at the individual level, aggregated for groups and conditions separately. Shaded traces represent the standard error of the mean. Vertical dashed lines indicate the number of components corresponding to the effective dimensionality of each group in each condition. (b) Effective dimensionality values for each participant, plotted separately for the local and global condition. Each data point reflects data at the participant level. (c) Group‐level distributions of effective dimensionality for younger and older participants, split by local and global networks. The violin plots display the smoothed distribution of the data, along with the individual values, the group means, and bars representing one standard deviation. No significant group or interaction differences were observed. Significance levels: * *p* < 0.05, ** *p* < 0.01, *** *p* < 0.001; The *p*‐value was significant after Bonferroni correction.

Across all groups and conditions, the cumulative variance explained increased steeply for the first principal components before gradually approaching an asymptote (Figure 6a), indicating that a limited subset of components accounted for the dominant whole‐brain signal. However, the rise in explained variance was consistently delayed in the global compared to the local condition. This pattern suggests that processing global stimulus regularities involved a larger set of orthogonal network components.

At the individual level, ED varied substantially across participants (Figure 6b). Nevertheless, ED was higher in the global condition (M = 5.67, SD = 1.40) than in the local condition (M = 3.94, SD = 1.12), with 71 out of 77 participants showing this pattern. The within‐subject difference (global − local) showed a mean of 1.74 (SD = 1.37).

A 2 × 2 mixed ANOVA with Condition (Local, Global) as a within‐subject factor and Group (Younger, Older) as a between‐subject factor revealed a robust main effect of Condition, F(1,75) = 121.34, *p* < 0.001, partial η^2^ = 0.62, indicating substantially higher ED in the global condition. The effect size was large, suggesting that approximately 62% of the explainable within‐subject variance was attributable to condition differences. There was no evidence for a main effect of Group, F(1,75) = 0.08, p = 0.779, partial η^2^ = 0.001, nor for a Group × Condition interaction, F(1,75) = 0.08, p = 0.780, partial η^2^ = 0.001. Thus, the increase in ED from local to global conditions was comparable between younger and older adults (Figure 6c). Condition effects remained significant after Bonferroni correction (*p* < 0.001), while Group and Group x Condition effects remained insignificant (p = 1 in both cases). Although Anderson–Darling tests indicated deviations from normality in the local condition within each age group, the mixed ANOVA is generally robust to moderate non‐normality in balanced designs. Moreover, the effect of Condition was highly consistent across participants, supporting the stability of the observed effect.

Together, these findings indicate that the local condition engaged more compact and constrained network configurations, whereas the global condition required a broader and more distributed set of network components to explain the variance of the data. This pattern is consistent with the idea that the processing of global deviations recruits more widely distributed large‐scale connectivity patterns relative to the processing of local deviations, although these analyses alone cannot determine whether these differences arise from changes in network strength, complexity, noise structure, or a combination of factors.

### 2.6 Investigating BROAD‐NESS Networks in Group and Condition Separated Data

**FIGURE 7:**
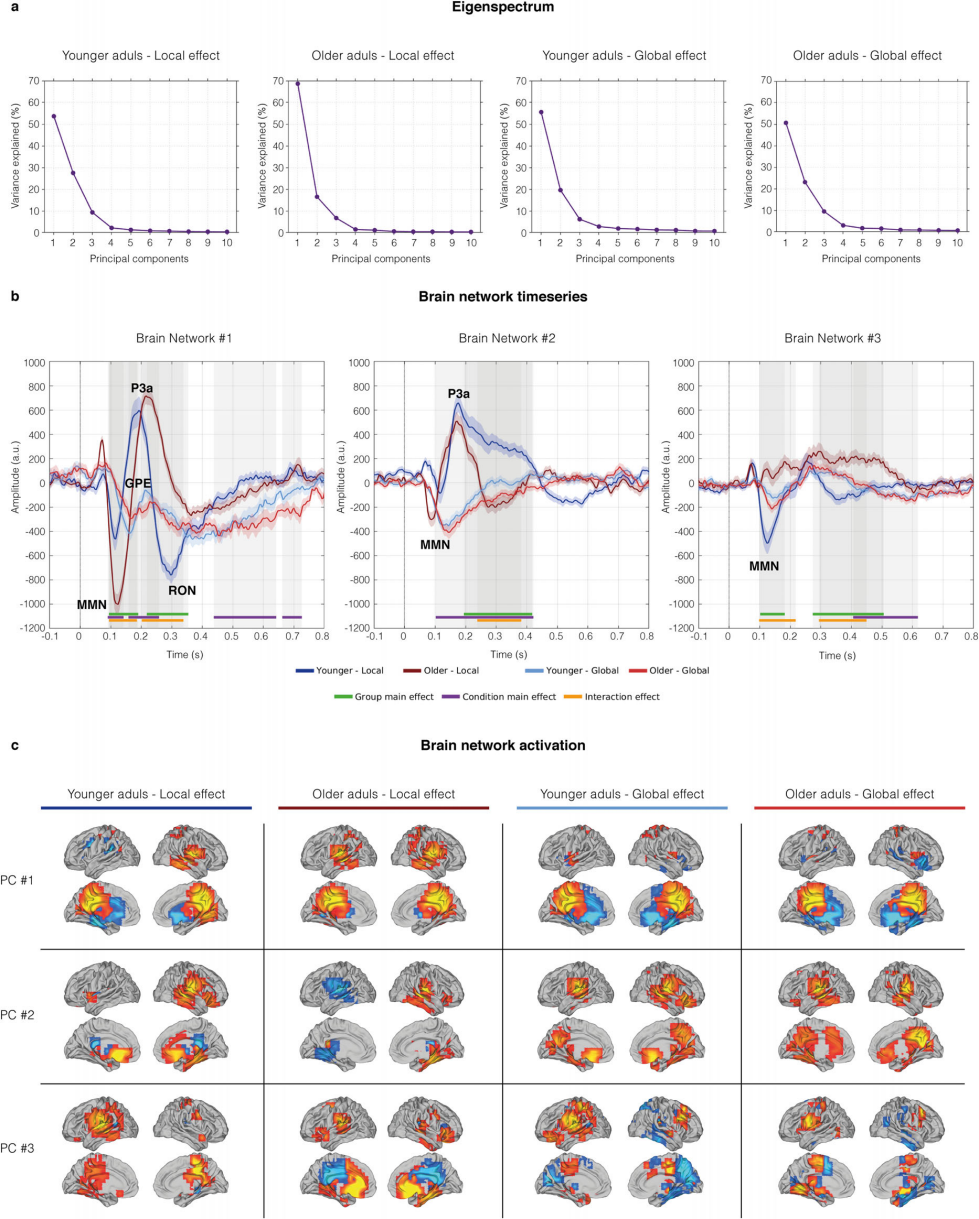
Time series and brain network activation patterns when BROAD‐NESS is applied to group‐ and condition‐separated data. The figure illustrates the eigenspectra, time series, and spatial activation patterns of brain networks when the BROAD‐NESS framework is applied to group‐ and condition‐separated data, which provides unique activation matrices for each group‐condition‐separated dataset. (a) Eigenspectra for group‐ and condition‐separated data. Each plot displays the variance explained by the first 10 principal components. Across all conditions, the first three components account for most of the variance, indicating that only a small number of components capture most of the signal structure. (b) Time series of the brain networks obtained from the first three principal components (PC #1–PC #3) from group‐ and condition‐separated data. Shaded areas indicate standard errors of the mean. Significant group, condition, and interaction effects are indicated by green, purple, and yellow lines. Time = 0 s. indicates the onset of the fifth tone in the local‐global paradigm. (c) Spatial activation maps of the brain networks obtained from the first three principal components for group‐ and condition‐separated data. The brain templates illustrate the spatial extent of the networks, with yellow voxels contributing the most and light blue voxels contributing the least to the time series. Only voxels with values exceeding the mean by more than one standard deviation in absolute terms are depicted. The weights of the spatial activation patterns were normalized independently for each PC.

To evaluate whether the time series and activation patterns identified by the BROAD‐NESS methodology were robust to other grouping and averaging choices, we repeated the PCA decomposition on activation matrices generated separately for each age group in each condition. This procedure yielded one activation matrix per group‐condition combination, allowing us to examine eigenspectra, time series, and spatial network structure under more constrained data partitions, providing further insights into potential group‐ and condition‐related differences. Across all datasets, the eigenspectra (Figure 7A) showed that the first few principal components accounted for the majority of variance in the data, with sharp drops after the first three components. This pattern suggests that a relatively low‐dimensional set of network processes captures most of the signal structure, even when PCA is performed on group‐ and condition‐separated data.

Inspection of the time series from the brain networks identified by the first three components revealed systematic age‐ and condition‐related modulations (Figure 7B). Brain Network #1 showed that tendencies in group, condition, and interaction effects remained the same as in the main analysis, revealing pronounced early local responses in older adults (MMN, P3a) and an enhanced later local RON response among younger adults. Brain Network #2 showed stronger local P3a responses in younger adults relative to older adults, demonstrating the same pattern observed in the main analysis. Brain Network #3 reflected increased MMN activity followed by later negative deflections, again with clearer expression in the local condition. Significant main effects of group and condition, as well as their interactions, occurred primarily within windows corresponding to ERF responses, and were more frequent in the local condition, suggesting a condition‐specific selectivity in network engagement. See Table S9 for specific results.

Spatial maps of the first three components further highlighted meaningful differences across groups and conditions (Figure 7C). Brain Network #1 involved bilateral coactivation of Medial and Posterior Cingulate cortices, Precuneus and Parietal areas, closely resembling the results of the main analysis. Interestingly, this coactivation differed among age groups in the local condition, as older adults showed connections to the auditory cortices of both hemispheres, while younger adults predominantly utilized the right hemisphere. Brain Network #2 in the local condition captured activation of the right Caudate, Insula, Rolandic Operculum, and Fusiform gyrus in both groups, while younger adults showed a stronger frontal engagement of the right Rectus, Anterior Cingulate Cortex, and Olfactory Bulb. In the global condition, younger adults activated similar regions as in the local condition, while these regions were also connected to bilateral Precuneus activation. A similar pattern was observed for older adults, showing a brain network connecting the auditory cortices to both frontal ACC and Rectus while also activating Precuneus and Medial/Posterior Cingulate Cortices. In Brain Network #3, younger adults displayed activation of left temporo‐central areas including pre‐ and post‐central areas, Cingulate cortices, and the left temporal lobe. Older adults displayed activation of the left insula and Caudate areas across conditions and a very pronounced frontal activation in the local condition, connecting bilateral Rectus, ACC, and Olfactory Bulb areas to Caudate and Fusiform areas. See Tables S10 and S11 for MNI coordinates and voxel counts within each brain area.

In sum, these analyses demonstrate that the time series and neural activation of the BROAD‐NESS networks remain largely robust when PCA is applied to group‐ and condition‐specific data. Though general tendencies in ERF responses and overall brain network structures remain consistent with the main analysis, this analysis also shows nuances in specific group‐ and condition‐related differences in the time series and underlying brain network activation.

## 3 Discussion

### 3.1 Summary

In the present study, we applied BROAD‐NESS, a PCA‐based broadband network decomposition framework, to source‐reconstructed MEG data acquired from the auditory local‐global paradigm, where both sensory and pattern violations occur. Our two primary aims were: (i) to characterize the neural responses associated with hierarchical prediction error signaling within large‐scale brain networks and their multivariate embedding in phase space. (ii) To examine how these processes are altered in healthy aging. Three dominant orthogonal brain networks obtained from the first three principal components accounted for 91.5% of the variance in the data and were robustly recoverable across alternative PCA implementation strategies and robustness analyses. Three key findings emerged in the study. First, automatically elicited neural responses associated with prediction error signaling toward sensory deviancy were enhanced in aging and relied on a primary network connecting bilateral auditory regions to the Medial and Posterior Cingulate Cortices. These regions are strongly associated with the recruitment of attentional neural resources. Conversely, neural responses indexing the reorientation of attention following sensory violations and prediction error responses from global pattern violations were attenuated with age. Second, the processing of local violations involved a more recurrent and deterministic recruitment of the three main brain networks across time, while the processing of global violations was more divergent and exploratory. Third, the processing of global pattern violations was characterized by higher effective dimensionality of principal components. This suggested involvement of a wider range of orthogonal brain networks across the brain, compared to local violation processing, which was more confined to fewer brain networks.

Together, these findings may nuance existing predictive coding models of aging, where predictive processes indexed by ERF responses are expected to uniformly attenuate in strength independent of task demands. Instead, the present findings suggest (i) that sensory‐based neural processes associated with predictive processing can enhance with age, while more cognitive processes attenuate, (ii) that the sensory‐based processes are rooted in a large attention‐related cingulo‐parietal network, and (iii) that the dynamical distinctions between local and global processing are preserved with age. Below, we discuss each major set of results in turn, situating them within predictive coding and aging theoretical frameworks, while also considering alternative interpretations and methodological limitations.

### 3.2 Large‐Scale Network Organization of Hierarchical Deviance Responses

We applied BROAD‐NESS to source‐reconstructed MEG data and revealed three significant PCs that accounted for 51.78%, 24.57%, and 15.12% of the variance in the data and displayed distinct network characteristics. Brain Network #1 recruited bilateral auditory cortices including Heschl's gyri, Medial and Posterior Cingulate Cortices, and Precuneus areas. These Parietal regions have repeatedly been implicated in attentional monitoring, salience regulation, and integrative cognitive control [52]. In contrast, Brain Network #2 and #3 were characterized by a pronounced involvement of the left and right auditory cortices and bilateral frontal regions including ACC and Rectus. This organization suggests that the auditory cortex participates in multiple concurrent network configurations. In one configuration, the auditory cortices are connected to cingulo‐parietal regions and are likely involved in both the detection of violations and the updating of internal models of the environment–as both MMN and P3a were prominent within this network. In another configuration, the auditory regions of each hemisphere are connected to frontal areas including anterior cingulate circuits. Only the P3a response was prominently elicited within these networks, which aligns with previous studies reporting that the ACC is one of the neural generators of the P3a response [32, 41].

Interestingly, these findings reveal both convergences and divergences with recent work on predictive processes during working memory [53] and long‐term memory encoding [54] and during recognition of auditory sequences [9, 55, 56, 57, 58]. Similar large‐scale networks have been reported, involving auditory cortices, Medial and Anterior Cingulate regions, Hippocampus, Insula, and fronto‐opercular areas. However, in contrast to those studies, the present results indicate a functional dissociation between left and right hemispheres, as reflected in the separation of Brain Network #2 and #3. Such hemispheric differentiation was not observed in studies of conscious recognition, which instead showed a higher relative reliance on the Medial Cingulate gyrus [45]. This pattern suggests that while a common set of regions underpins neural responses associated with predictive processing across conscious and pre‐attentive contexts, their network configuration may differ depending on cognitive demands, particularly with respect to hemispheric specialization and relative involvement of the Medial Cingulate.

Finally, to further characterize the spatial organization of these networks, we embedded voxel‐wise activation coefficients on each of the three brain networks within a 3D gradient space and applied k‐means clustering. A 16‐cluster solution revealed spatially coherent and anatomically interpretable voxel groups. While several clusters contributed positively to one or two brain networks, no cluster showed contributions to all three networks. This illustrates how the three BROAD‐NESS networks capture distinct but partially overlapping patterns of brain network co‐involvement, embedded in continuous gradients rather than sharply segregated modules.

### 3.3 Aging‐Related Alterations in Hierarchical Deviance Processing

Our primary aging‐related finding concerned the pronounced alterations in ERF responses observed with age and the underlying brain networks supporting these processes. Older adults exhibited enhanced MMN‐ and P3a responses within an auditory‐cingulo‐parietal network. This increase in automatically elicited neural responses associated with sensory‐based prediction error signaling may indicate an increase in the processes involving the detection of sensory deviancy and the updating of internal models of sensory information. Conversely, more cognitively demanding ERF responses such as the late local RON response and the GPE response showed age‐related attenuation within this network. This suggests a diminished attentional reorientation toward the paradigm (RON) and neural response toward pattern violations (GPE) in older adults.

Together, this pattern is consistent with results from a recent EEG study utilizing the local‐global paradigm, where older adults show no differences in MMN and P3a while the GPE response and RON responses were attenuated and predictive of future working memory decline [21, 59]. The GPE response has previously been associated with conscious context updating and working‐memory revision, elicited when a larger contextual pattern is violated, with generators spanning parietal, cingulate, and frontoparietal networks [32, 60]. Age‐related reductions in the amplitude of the GPE response have therefore commonly been attributed to impairments in higher‐order contextual integration and working‐memory updating [21, 30]. Similarly, the RON response involves cognitively demanding processes to reorient attention toward the paradigm after distractions from sensory deviancy [40, 61]. This suggests an age‐related difference in the recruitment of neural resources during complex tasks, where sensory‐based processes are being prioritized ahead of more cognitively demanding processes. Furthermore, in a complex auditory memory paradigm involving the same participants as in the present study, an age‐related enhancement of both an early negative response and later, slower positive components was observed [62]. This pattern was interpreted as reflecting a compensatory mechanism involving increased recruitment of attentional resources, which may contribute to the partial preservation of auditory and general memory abilities observed in this and similar cohorts [63, 64]. Taken together, these results suggest that in complex tasks, age‐related enhancements of processes involved in sensory deviancy detection may be enhanced through attention‐related Medial and Posterior Cingulate brain network involvement.

At face value, this pattern challenges the canonical scalp‐level literature predominantly reporting an age‐related attenuation of neural processes associated with prediction error signaling toward sensory violations [18, 65, 66]. Rather than a uniform reduction, our results suggest that aging involves a reallocation of attention‐related neural resources, enabling enhancements of automatic, sensory‐based prediction error responses – potentially at the cost of more cognitive processes involved in conscious working memory updating and reorientation of attention toward the paradigm. This general pattern of finding increased sensory responses and attenuated cognitive responses in older adults may very well depend on utilizing an active paradigm, where attention is directed toward a single deviating tone. In previous studies comparing responses from the local‐global paradigm across age, attention was directed toward pattern‐level deviances, which may explain why these studies find no age‐related differences in the local MMN response [21, 59]. Both the results of the present study and the previous studies investigating hierarchical effects in older adults indicate that the uniform decline account should be revised, as early sensory responses like MMN can appear unaffected or even increased with age depending on the paradigm. Contrary to the traditional passive oddball paradigms, where MMN responses are often, but not consistently, found to decrease with age [16], enhanced MMN responses are observed in older adults in the present study, where attention is actively directed toward the sensory level. Furthermore, this pattern is consistent with the notion that older adults remain sensitive to sensory violations but show reduced capacity for sustained attentional reorientation and processing of global violations, as these processes are strongly dependent on working memory functions [41, 59].

In addition, these processes may be supported by different network configurations in aging. When specifically examining age‐related differences when applying BROAD‐NESS to group‐ and condition‐separated data, the primary brain network (PC #1) showed connections between bilateral auditory cortices and cingulo‐parietal regions in older adults – whereas younger adults exclusively displayed connections between the right auditory cortex and cingulo‐parietal regions. This supports the Hemispheric Asymmetry Reduction in Older Adults model (HAROLD), where aging is theorized to reduce lateralization in favor of a bilateral recruitment of networks across hemispheres [67]. This could suggest an age‐related reduction in the hemispheric specialization of neural circuits, where bilateral auditory cortices rather than the right auditory cortex are connected with central Cingulate cortices to process sensory violations.

### 3.4 Stimulus‐Specific Adaptation as an Alternative Explanation

A critical interpretive challenge concerns the potential contribution of stimulus‐specific adaptation (SSA) to age‐related differences in early deviance responses. SSA is ubiquitous in the auditory cortex and contributes to MMN generation alongside predictive mechanisms [68, 69]. Importantly, SSA is known to decline with age [70, 71], raising the possibility that group differences in MMN amplitude may partly reflect decreased neural adaptation rather than enhanced prediction error signaling. The local‐global paradigm is inherently repetitive, which increases susceptibility to adaptation confounds. If older adults exhibit reduced adaptation to deviant tones, MMN‐like difference waves may be amplified even in the absence of enhanced prediction error computation. This issue has been highlighted in critiques of the paradigm's reliability in eliciting canonical MMN responses [72], while in recent work a limited correspondence between MMN measures derived from oddball vs. local‐global designs has been shown [73]. Thus, the observed age‐related enhancement of early components within Brain Network #1 could reflect adaptation‐related variance becoming more globally distributed rather than compensatory recruitment of attention‐related neural cortices. However, SSA effects are not deemed likely beyond the MMN response for two central reasons: (i) An age‐related enhancement of P3a is observed, which is not known to be modulated by SSA. (ii) The dominant involvement of cingulo‐parietal regions in Brain Network #1, where SSA is not known to be present, suggests involvement of attention‐related neural resources. Despite these counterarguments, future work should incorporate paradigms designed to minimize SSA, including, e.g., roving standards, increased stimulus variability, or omission deviants to disentangle adaptation from predictive mechanisms.

### 3.5 Dynamical Regimes Depend on the Hierarchical Level of Deviance Processing

Beyond analyses of ERF responses, BROAD‐NESS enabled a dynamical systems characterization of brain network trajectories. Phase‐space embeddings of network time series revealed that local deviants elicited more recurrent trajectories, whereas global deviants induced more dispersed trajectories. Recurrence quantification analysis confirmed robust condition effects across all included metrics, with local processing showing higher recurrence, determinism, trapping time, diagonal length, maximal vertical line length, laminarity, and entropy, while global processing showed higher divergence. These findings are theoretically consistent with hierarchical predictive coding accounts. Local deviants violate short‐lived sensory regularities and can be processed through relatively confined, recurrent circuits that give rise to easily identifiable and pronounced ERF responses. Global deviants require integration over longer time scales, revision of higher‐order predictions, and engagement of conscious access mechanisms associated with the activation of global workspace systems across wide‐ranging brain networks within the cortex [7, 42, 43, 74, 75, 76, 77]. Furthermore, ERF responses elicited toward global pattern violations are less pronounced and have a higher latency [7]. Importantly, these dynamical distinctions were largely age‐invariant, suggesting that aging remodels the spatial embedding of deviance responses but does not abolish the qualitative dynamical separation of local and global deviance processes associated with prediction error signaling.

### 3.6 A Larger Network Organization Observed in Global Deviance Processing

Similarly, the ED analysis provided converging evidence that the processing of global pattern violations engages a broader network recruitment across the brain. The processing of global deviants required a significantly increased ED across participants, indicating that more orthogonal components were required to account for signal variance. This aligns with the consistent findings that contextual violations recruit more distributed integrative systems consistent with global workspace theories and the conscious working memory‐dependent mechanisms involved in processing pattern violations [7, 43]. However, ED is ultimately a covariance‐based index rather than a direct measure of brain network complexity. Higher ED could reflect greater engagement of task‐relevant networks but also increased neural noise or strategy heterogeneity. Thus, we interpret the ED increase as evidence for broader variance distribution during global processing without equating it unambiguously with more sophisticated computation.

However, these findings contribute to an ongoing debate regarding the neural implementation of prediction error signaling in the local–global paradigm. Classical interpretations have often emphasized a hierarchical distinction in which local violations are processed primarily within auditory cortex, whereas global violations recruit frontal or frontoparietal regions responsible for higher‐order contextual processing. While our findings broadly agree with this hierarchical organization, they further suggest that global deviance processing is better understood as the coordinated engagement of distributed large‐scale brain networks rather than activity arising from a single frontal locus. Although frontal regions formed important components of Brain Networks #2 and #3, they consistently operated together with bilateral auditory cortices and temporo‐parietal regions, while the increased effective dimensionality observed during global violations indicated the recruitment of a broader repertoire of orthogonal network configurations than during local violations. This interpretation is consistent with recent work showing that hierarchical prediction and prediction‐error signals emerge through interactions among distributed cortical areas rather than isolated regional responses [78]. Likewise, recent information‐theoretic analyses demonstrate that prediction‐error representations are synergistically distributed across auditory and frontal cortices, with complementary information emerging specifically from interactions between regions rather than from individual areas alone [79]. Recent human MEG work further suggests that prediction‐error signaling supports adaptive changes in distributed representational geometry across temporal and frontoparietal networks during statistical learning [80], reinforcing the view that predictive processing is fundamentally a network‐level phenomenon.

Our findings may also be relevant to the recent proposal that global oddball responses may reflect a more cognitive process than originally envisaged by classical predictive‐coding models, potentially emerging predominantly within higher‐order frontal systems rather than constituting canonical prediction‐error signals throughout the cortical hierarchy [81]. While the MEG data of the present study cannot distinguish whether the observed distributed responses primarily reflect local computations or afferent inputs to these regions, they nevertheless demonstrate that frontal engagement during global deviance occurs as part of widespread, coordinated cortical networks rather than in isolation. Importantly, this distributed architecture appeared largely preserved with aging. Although older adults showed selective attenuation of later cognitive responses and enhancement of earlier sensory‐related responses, neither the recurrence analyses nor the effective‐dimensionality analyses suggested a collapse of the distinction between local and global processing. Global violations continued to recruit broader and more divergent network configurations than local violations in both age groups. Together, these findings suggest that healthy aging primarily alters the relative engagement of individual network components rather than disrupting the large‐scale distributed architecture supporting hierarchical prediction‐error processing.

### 3.7 Limitations and Future Directions

Four limitations should be acknowledged. First, the study was cross‐sectional, preventing direct inference about within‐individual aging trajectories. Second, SSA remains a plausible confound for early component differences, motivating future meticulous manipulations of the stimuli incorporated within local‐global paradigms. Third, detailed audiometric thresholds were not obtained in the study, despite their ideal utility as covariates in statistical analyses. Fourth, frequency‐specific analyses may provide enhanced mechanistic traction, given predictive coding theories that link distinct oscillatory bands to top‐down and bottom‐up signaling [2, 82]. Future work should therefore aim to address these aspects to further advance knowledge on age‐related changes in neural responses associated with hierarchical prediction error signaling.

## 4 Conclusion

In summary, the results of the present study suggest that aging does not uniformly attenuate neural responses associated with hierarchical prediction error signaling. Rather, in complex tasks, the aging brain is capable of enhancing sensory‐based processes of individual deviating stimuli, while more cognitively demanding processes, involved in pattern processing and in the reorientation of attention, are attenuated. By applying the PCA‐based BROAD‐NESS methodology to source‐reconstructed MEG data from the auditory local–global paradigm, we identified three concurrent whole‐brain networks supporting neural responses associated with predictive processing. In older adults, an enhancement of neural responses associated with local prediction error signaling was observed within a primary brain network connecting cingulo‐parietal areas to the auditory system of the temporal lobes. These enhancements were exclusive to early, sensory‐based processes among older adults, while cognitive processes involved in the reorientation of attention and working memory functions were attenuated. While these findings may likely be explained by a predictive coding account, SSA remains ubiquitous in the auditory cortex and could potentially influence the age‐related enhancement of early neural responses elicited from sensory violations. Crucially, the dynamical distinction between local and global processing remained preserved, with global violations engaging broader, higher‐dimensional network dynamics. These findings underscore the importance of whole‐brain network approaches in potentially refining theoretical models of aging, as found in predictive coding, while also elucidating age‐invariant dynamical aspects embedded in the processing of sensory and pattern violations.

## 5 Methods

### 5.1 Participants

The sample comprised 77 participants (43 females), who were distributed in two age groups: (i) A younger adult group comprising 37 participants (18 females) aged 18 to 25 years (mean age: 21.89 ± 2.05 years); (ii) an older adult group comprising 40 participants (24 females) aged 60–81 years (mean age: 67.50 ± 5.46 years). All participants held Danish nationality. Inclusion criteria encompassed: (i) overall good health with no reported neurological or psychiatric issues, (ii) chronological age of either 18–25 years or 60 years and above, (iii) normal hearing adjusted to age, (iv) normal or corrected‐to‐normal vision (e.g., via contact lenses), and (v) a clear understanding of and agreement to the provided participant information. Exclusion criteria encompassed: (i) use of prescribed medication affecting the central nervous system, (ii) neurological or psychiatric conditions, (iii) unwillingness to cooperate or verbally agree to participation, (iv) MRI contraindications, (v) being 26–59 years old, and (vi) impaired hearing. The Institutional Review Board of Aarhus University, Denmark, approved the project (case number: DNC‐IRB‐2021‐012). All experimental procedures were in compliance with the Declaration of Helsinki – Ethical Principles for Medical Research (World Medical Association Declaration of Helsinki, 2013). All participants were informed about the purpose of the study before giving consent to participate. A brief demographic questionnaire was administered prior to MEG data collection, obtaining information regarding age, highest level of achieved education, and biological sex. See Table 1 for an overview.

**TABLE 1:** Demographic information. Data are reported in whole numbers or mean ± SD.

	Younger adults (n = 37)	Older adults (n = 40)	*p*‐values
Sex (females/males)	18 / 19	24 / 16	—
Age in years	21.89 ± 2.05	67.50 ± 5.46	<0.001***
Years of education	13.84 ± 1.85	14.88 ± 3.07	0.074

### 5.2 Experimental Paradigm

An adapted version of the auditory local‐global paradigm was used [7]. The task consisted of 160 trials per block for a total of four blocks. Each block consisted of a combination of patterns consisting of either five repetitive tones or patterns where the fifth tone deviated in pitch (Figure S1). Two types of deviating fifth tones were used – an experimental deviating tone to induce a local sensory violation, and a deviating target tone to ensure continued attention toward the paradigm, as participants were instructed to press a button upon hearing this tone.

The local‐global paradigm was designed to elicit neural responses at two hierarchical levels: Canonical local neural responses were expected to be elicited from sensory violations at the individual tone level, while canonical global neural responses were expected to be elicited from violations at the pattern level. Both congruent and incongruent blocks were used (Figure S1). In congruent blocks, local standard trials with five repetitive tones occurred in 75% of experimental trials, while local deviant trials with a fifth deviating experimental tone occurred in 25% of experimental trials. In both congruent and incongruent blocks, target tones were elicited 10 times in addition to the experimental trials to ensure continued attention toward the paradigm. In incongruent blocks, local standard trials switched probability of occurrence with local deviant trials to create a global violation regarding the frequency with which stimuli were presented. The ordering of congruent and incongruent blocks was randomized among participants. The duration of each of the five tones in each trial was 80 ms, followed by 20 ms of silence after each tone, yielding a stimulus onset asynchrony of 100 ms within patterns. Between the five tone patterns, the intertrial interval was 500 ms. Three pitch levels were used, centered around 440 Hz and spaced in semitone steps (−4, 0, +10 semitones). The local‐global paradigm concluded after all four blocks, lasting 13 min and 20 s excluding pauses, while the entire MEG session concluded after approximately 1 h, as other tasks were also administered. The order of tasks in a session was randomized across participants. The global‐local‐paradigm was presented using PsychoPy version 2023.2.3 [83].

Instructions were displayed visually on a screen before the experiment. Participants were instructed to listen attentively to the tone sequences, respond as quickly as possible to the high‐pitched target tones, and maintain fixation on a centrally presented fixation cross throughout the experiment. Instructions prompted participants to identify the target tone in each block by pressing a button on a joystick. A practice phase familiarized participants with detecting the target tone. Practice continued until participants produced three correct responses occurring within 2 s following target onset during a practice sequence of 20 trials, ensuring task comprehension before continuing. Upon completing the practice phase, two congruent and two incongruent blocks were presented in random order. Auditory stimuli were delivered through a speaker placed 2 meters in front of the subject at a sound pressure level of ∼60 dB for 68 participants. For 9 participants, sounds were presented at ∼70 dB, as they were 70 years or older and showed mild hearing impairment, typically associated with aging. Only auditory stimuli in the 125–650 Hz range were used, as these frequencies are minimally impacted by typical age‐related hearing loss [84]. Event timing, response times, and task events were logged for offline analyses.

### 5.3 Neural Data Acquisition

The MEG recordings were conducted in a magnetically shielded room at Aarhus University Hospital (AUH), Denmark, using an Elekta Neuromag TRIUX MEG scanner with 306 channels (Elekta Neuromag, Helsinki, Finland). Data were collected at a sampling rate of 1,000 Hz with an analog filtering of 0.1–330 Hz. Prior to the recordings, participants’ head shapes and the positions of four Head Position Indicator (HPI) coils were registered relative to three anatomical landmarks using a 3D digitizer (Polhemus Fastrak, Colchester, VT, USA). This information was later used to co‐register the MEG data with anatomical MRI scans. Throughout the MEG recording, the HPI coils continuously tracked the position of the head, allowing for movement correction during analysis. Additionally, two sets of bipolar electrodes were used to monitor cardiac rhythm and eye movements, facilitating the removal of electrocardiography (ECG) and electrooculography (EOG) artefacts in pre‐processing.

MRI scans were obtained on a CE‐approved 3T Siemens MRI scanner at AUH. The data included structural T1 (MPRAGE with fat saturation) scans, with a spatial resolution of 1.0 × 1.0 × 1.0 mm and the following sequence parameters: echo time (TE) of 2.61 ms, repetition time (TR) of 2300 ms, a reconstructed matrix size of 256 × 256, an echo spacing of 7.6 ms, and a bandwidth of 290 Hz/Px. The MEG and MRI recordings were conducted on separate days.

### 5.4 MEG Data Pre‐Processing

The raw MEG sensor data (comprising 204 planar gradiometers and 102 magnetometers) was initially pre‐processed using MaxFilter [85] (version 2.2.15) to reduce external interference. We applied signal space separation (SSS) with the following MaxFilter parameters: downsampling from 1,000 to 250 Hz, movement compensation via continuous HPI coils (default step size: 10 ms), and a correlation limit of 0.98 to reject overlapping signals between inner and outer subspaces during spatiotemporal SSS.

The MEG data was converted into Statistical Parametric Mapping (SPM) format and further processed and analyzed using MATLAB version R2024b (MathWorks, Natick, MA, USA), incorporating custom‐built scripts (LBPD, https://github.com/leonardob92/LBPD‐1.0.git) and the Oxford Centre for Human Brain Activity (OHBA) Software Library (OSL) [86] (https://ohba‐analysis.github.io/osl‐docs/), which integrates Fieldtrip [87], FSL [88], and SPM [89] toolboxes.

Next, the continuous MEG data underwent visual inspection via the OSLview tool to remove any large artifacts, and less than 0.1% of the collected data was discarded. Independent Component Analysis (ICA) was then used (with OSL implementation) to eliminate eyeblink and heartbeat artifacts [90]. The ICA process involved decomposing the original signal into independent components and correlating these components with the activity recorded by the electrooculography (EOG) and electrocardiography (ECG) channels [91]. Components that showed a correlation at least three times higher than others were flagged as reflecting EOG or ECG activity. These flagged components were further validated through visual inspection, ensuring their topographic distribution matched typical eyeblink or heartbeat activity, and were subsequently discarded. The signal was then reconstructed using the remaining components. The signal was epoched using a 100 ms baseline before the onset of the fifth tone and 800 ms following this onset. To achieve an equivalent signal‐to‐noise ratio between standards and deviants, an equivalent number of standard epochs were randomly selected. For each group and for each condition, signals from standard and deviant epochs were averaged separately before being subtracted from each other.

### 5.5 Source Reconstruction

MEG is a powerful tool in detecting whole‐brain activity with excellent temporal resolution. However, to fully understand the brain activity involved in complex cognitive tasks, it is essential to identify the spatial sources of this activity. This requires solving the inverse problem, as the MEG recordings reflect neural signals from outside the head but do not directly indicate which brain regions generated them. To address this, we employed beamforming algorithms [92, 93, 94], using a combination of in‐house‐developed codes alongside the OSL, SPM, and FieldTrip toolboxes. The procedure consisted of two main steps: (i) designing a forward model and (ii) computing the inverse solution.

First, a single‐shell forward model was constructed using an 8 mm grid. This head model treats each brain source as an active dipole and describes how the activity of such a dipole would be detected across MEG sensors [93, 95]. The MEG data were co‐registered with individual T1‐weighted MRI scans, using the 3D digitizer information to align the data. When individual anatomical scans were unavailable, an MNI152‐T1 template with 8 mm spatial resolution was used to compute the forward model.

Second, a beamforming algorithm was applied as the inverse model. Beamforming uses a set of weights applied to different source locations (dipoles) to isolate the contribution of each brain source to the activity recorded by the MEG sensors [92, 93, 96]. This was done for every time point of the recorded brain data, allowing us to reconstruct the spatial sources of the MEG signal. The detailed steps for implementing the beamforming algorithm are provided below.

The data recorded by the MEG sensors (*B*) at time *t* can be described by the following Equation (1):

(1) $$B_{\left(t\right)}=L*Q_{\left(t\right)}+\varepsilon$$

where *L* is the leadfield model, *Q* is the dipole matrix carrying the activity of each active dipole (*q*) at time *t*, and *Ɛ* is noise (for details, see Huang et al. [93]). To solve the inverse problem, *Q* must be estimated for each *q*. In the beamforming algorithm, a series of weights are computed to describe the transition from MEG sensors to the active dipole *q*, independently for each time point. This is reported in Equation (2):

(2) $$q_{\left(t\right)}=W^{T}\;*B_{\left(t\right)}$$

Here, the superscript *T* refers to the transpose of a matrix. To compute the weights (*W*), matrix multiplication between *L* and the covariance matrix of MEG sensors (*C*) is performed. Importantly, the covariance matrix *C* was computed on the signal after concatenating the single trials of all experimental conditions. For each dipole *q*, the weights (*W_q_*) were computed as shown in Equation (3):

(3)

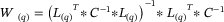




The computation of the leadfield model *L* was performed for the three main orientations of each dipole [95]. Before computing the weights, to simplify the beamforming output [97, 98], the orientations were reduced to one using the singular value decomposition algorithm on the matrix multiplication reported in Equation (4):

(4) $$L=svd{\left(l^{T}*\;C^{-1}*l\right)}^{-1}$$

Here, *l* represents the leadfield model with the three orientations, while *L* is the resolved one‐orientation model that was utilized in Equation (3).

Once computed, the weights were normalized for counterbalancing the reconstruction bias toward the center of the head [92, 99, 100, 101] and applied to the neural activity averaged over trials, independently for each time point (Equation 2) and experimental condition. This procedure returned a time series for each of the 3,559 brain sources [92, 100].

### 5.6 Principal Component Analysis (PCA)

PCA was applied to the time series of the 3,559 reconstructed brain sources to disentangle broadband brain networks operating simultaneously. PCA is a dimensionality reduction method that transforms data into new variables, or principal components, which account for the most variance within the dataset. This is achieved by computing the eigenvectors and eigenvalues from the covariance matrix of the data and then projecting the data onto the directions of maximum variance. Traditionally, PCA simplifies high‐dimensional data while retaining the most important information.

In this study, however, PCA was applied to a dense set of MEG‐reconstructed brain data at the voxel level (3,559 brain voxel time series). The goal was not just to reduce the dimensionality but to identify brain networks obtained from PCs involved through the application of PCA on the 3,559 brain voxel time series.

In short, PCA operates as follows. First, the data is centered by subtracting the mean of each brain voxel time series from the dataset *X*, as represented by Equation (5):

(5) $$\bar{X}=X-\mu$$

where μ is the mean vector of *X*.

Then, the covariance matrix *C* is computed on the centered data, as shown in Equation (6):

(6) $$C=\frac{1}{n-1}\;{\bar{X}}^{T}\bar{X}$$

where *n* is the number of data points.

Then the eigenvalue equation for the covariance matrix is solved to find eigenvalues and eigenvectors, Equation (7):

(7) CW=WΛ

where *W* is the eigenvector matrix (set of weights for each principal component) and Λ is the diagonal matrix of the corresponding eigenvalues (which indicate the amount of variance explained by each component).

Then, the eigenvectors *w* were used to compute the activation time series of each brain network *y* by multiplying them by the original data *X*, as shown in Equation (8):

(8) $$y=w^{T}X$$

Furthermore, we computed the spatial projection of each component (spatial activation patterns *a*) in brain voxel space. This was done by multiplying the weights of the analysis (the eigenvectors *w* in this case) by the covariance matrix *C*, as shown in Equation (9):

(9) $$a=w^{T}C$$

Interestingly, while previous studies on interpreting weights from multivariate analyses in MEG data have recommended calculating spatial activation patterns [102], in this case, the relative contribution of each brain voxel to the network remains the same, whether using the direct eigenvector *w* or computing the spatial activation patterns.

It is important to note that PCA can be computed using different mathematical approaches that yield equivalent results. A commonly used alternative is to apply Singular Value Decomposition (SVD) [103] directly to the mean‐centered data matrix. This approach is often preferred in practice because it is more numerically stable and computationally efficient, especially for high‐dimensional data (for instance, this is the solution currently implemented in the “pca” function in MATLAB). However, since the SVD‐based solution ultimately produces the same principal components and scores as the covariance‐based eigendecomposition we describe above, we chose to retain the latter formulation. This choice is motivated by clarity and pedagogical value, as it offers a more intuitive explanation of the underlying principles.

Importantly, as detailed in the following section, the PCA procedure was applied across various scenarios to ensure a comprehensive evaluation of the algorithm's performance. This included performing PCA in three different scenarios: (i) on data averaged across participants and conditions in the main analysis. (ii) On data from each participant in each condition in the brain network modularity analysis. (iii) On group‐ and condition‐averaged data as a robustness check and to elucidate more nuanced group‐ and condition‐related differences complementing the main analysis.

### 5.7 Statistical Analysis of ERF Responses

After computing PCA on the group‐averaged data, we extracted the time series of the resulting brain networks for each participant in each condition (Local, Global) [45]. Followingly, we assessed group‐ and condition‐related effects within a 2 × 2 mixed factorial framework, using separate permutation‐based tests to evaluate the Group main effect, Condition main effect, and Group × Condition interaction. For each of the first three brain networks, we restricted the analysis to the 0–800 ms post‐stimulus interval and performed cluster‐based permutation tests over time following the framework of Maris and Oostenveld [104]. At each time point, we tested (i) a between‐subject Group effect by comparing younger and older adults after averaging across conditions, (ii) a within‐subject Condition effect by contrasting the global vs. local condition across all participants, and (iii) a Group × Condition interaction defined as the difference in Global–Local contrasts between groups. Statistical inference was performed using cluster‐based permutation tests over time with 5,000 permutations, where cluster‐mass statistics controlled the family‐wise error rate. Clusters of neighboring supra‐threshold time points were first defined using a pointwise cluster‐forming threshold of *p* < 0.05. The resulting cluster masses (sum of absolute effect values) were then compared against a reference distribution of maximum cluster masses obtained under the null hypothesis by shuffling group labels for between‐subject effects and applying random sign flips for the within‐subject contrast. The family‐wise error rate was controlled at α = 0.05 based on this max‐cluster‐mass distribution. For each PC and each effect, we obtained binary significance masks and corresponding time windows of significant clusters, which were then used to visualize colored intervals indicating periods of significant group, condition, or interaction effects. All statistical analyses were implemented in MATLAB using the standardized routines described above and including the full sample of 77 participants.

### 5.8 Network‐Based ERF Latency Analysis

To characterize the temporal sequence of activity across large‐scale brain networks, participant‐specific ERF time series were extracted from the first three principal brain networks identified using the BROAD‐NESS framework. Source‐reconstructed MEG data were decomposed into principal components, yielding network time series for PC #1, PC #2, and PC #3 for each participant and condition. Analysis was restricted to the first three principal components, which represented the dominant spatial networks identified by the decomposition. First, mean latencies for each ERF response were identified by the latency of the most positive or negative peak for each group in each condition within characteristic intervals (Figure S2). These mean values for each age group in each condition were subsequently used to identify peak latencies for each participant, where specific time windows for each ERF response were defined as starting 50 ms before these mean latencies and ending 50 ms after them. Latencies were defined as the time point of the detected peak within this time window. Latencies were extracted separately for every ERF response for each participant in both conditions in each brain network.

To determine whether a consistent temporal ordering existed among the three networks, latency differences between PC #1, PC #2, and PC #3 were evaluated using Friedman tests, a non‐parametric repeated‐measures alternative to one‐way repeated‐measures ANOVA. Analyses were performed separately for each condition and each ERF response using complete‐case participant data. When significant omnibus effects were observed, pairwise Wilcoxon signed‐rank tests were conducted for PC #1–PC #2, PC #1–PC #3, and PC #2–PC #3 comparisons. Resulting *p*‐values were corrected for multiple comparisons using the Benjamini–Hochberg false discovery rate (FDR) procedure. The observed temporal ordering of networks was determined from mean latency estimates across participants.

### 5.9 Phase Space and Recurrence Quantification Analysis of PCA‐Derived Networks

To investigate the temporal dynamics and recurrence properties of the brain networks identified via the PCA‐based BROAD‐NESS methodology, we conducted a phase space and recurrence quantification analysis [47, 48] on the time series of the three main PCA components (brain networks). This approach allows for the examination of the brain's timewise utilization of the three main brain networks depicted in a phase space trajectory within a low‐dimensional temporal space, revealing underlying temporal structures that may not be evident in the original signal.

We first constructed 2D and 3D phase space trajectories by treating the time series of the first three BROAD‐NESS networks as independent axes in phase space. For each participant and experimental condition, the time‐resolved trajectory in this space was computed. Each point in the resulting scatterplot represents the state of the brain at a given time point, embedded in the space defined by the three dominant networks.

In order to statistically evaluate differences in phase‐space dynamics, we compared the mean trajectories of the first three PCA‐derived BROAD‐NESS networks in the 3D phase space from −100 to 800 ms post‐stimulus onset of the fifth tone.

To compare age groups in each condition (Local and Global), group‐average trajectories were computed separately for younger and older participants. At each time point, the separation between the two trajectories was quantified as the Euclidean distance in 3D phase space:

(10) $$D\;\left(t\right)=\left|\right|\mu_{younger}\left(t\right)-\mu_{older}\left(t\right)\;|{|}_{2}$$

where

(11) $$\mu_{young}\left(t\right)=\left.\left[PC_{1,\;young}\left(t\right),PC_{2,\;young}\left(t\right),PC_{3,\;young}\left(t\right)\right.\right]\;$$

and

(12) $$\mu_{older}\left(t\right)=\left.\left[PC_{1,\;older}\left(t\right),PC_{2,\;older}\left(t\right),PC_{3,\;older}\left(t\right)\right.\right]\;$$

represent the mean phase‐space coordinates of the younger and older groups, respectively. To assess statistical significance, a non‐parametric cluster‐based permutation test was performed across time (5,000 permutations). Under the null hypothesis of no age group difference, participant labels were randomly shuffled between groups while preserving the original group sizes. For each permutation, the Euclidean distance trajectory *D*(*t*) was recomputed, generating a null distribution at each time point. Time points exceeding the 95th percentile of the permutation distribution (cluster‐forming threshold α = 0.05) were grouped into contiguous temporal clusters. Cluster mass was calculated as the sum of Euclidean distances within each cluster:

(13) $$M=\sum D\left(t\right)$$

Cluster significance was determined by comparing observed cluster masses against the distribution of maximum cluster masses obtained from the permutations. Clusters were considered significant at a cluster‐corrected threshold of *p* < 0.05.

In order to compare Local and Global processing across all participants, mean trajectories were computed separately for the two conditions. Phase‐space separation was quantified as,

(14) $$D\;\left(t\right)=\left|\right|\mu_{local}\left(t\right)-\mu_{global}\left(t\right)\;|{|}_{2}$$

where µ_*local*_(*t*) and µ_*global*_(*t*) denote the mean 3D phase‐space coordinates of the Local and Global conditions, respectively.

Statistical significance was evaluated using a paired cluster‐based permutation procedure. For each permutation, Local and Global condition labels were randomly exchanged within participants, thereby preserving the paired structure of the data. The same cluster‐forming threshold (α = 0.05), cluster‐mass statistic, and maximum‐cluster correction procedure described above were applied. Significant clusters therefore indicate time periods during which the Local and Global conditions occupied significantly different regions of the 3D phase space.

After obtaining time windows of significant differences in both the age group and condition comparisons, the Euclidean distance was calculated from the mean of the group or condition to the midpoint of the phase space (0,0,0). The group or condition with the largest distance to the midpoint of the phase space was deemed the group or condition with the greatest excursion in phase space for each time point.

Following this, we calculated recurrence plots for each participant and condition. Recurrence plots reflect the degree to which the system re‐enters similar states over time [47, 48]. Specifically, for each pair of time points, we computed the Euclidean distance between their respective phase space coordinates. Recurrence matrices were thresholded using a fixed ε‐radius criterion (10% of the maximum distance) to obtain binary plots, where a value of 1 indicates a recurrence [47, 48]. From the thresholded recurrence plots, we extracted eight standard metrics commonly used in nonlinear time‐series and brain dynamics research. For full mathematical descriptions, see [48].

1. Recurrence Rate (RR): proportion of recurrent points, indexing overall recurrence of the system.
2. Mean Diagonal Line Length (L): average length of diagonals, indicating temporal predictability.
3. Trapping Time (TT): average vertical line length, reflecting temporal persistence in a given state.
4. Maximal Laminar Duration (Vmax): length of the longest vertical line (i.e., period of state stability).
5. Entropy (ENTR): Shannon entropy of diagonal lengths, indexing complexity of the system's evolution.
6. Determinism (DET): proportion of recurrence points forming diagonal structures, reflecting the stability of the system trajectories.
7. Laminarity (LAM): fraction of recurrence points forming vertical lines, reflecting intermittency.
8. Divergence (DIV): inverse of the longest diagonal line, linked to system sensitivity and divergence in state space.

These metrics were computed independently for each participant in each condition. Statistical analyses were performed using linear mixed‐effects models implemented in MATLAB. For each metric, we fitted a model with Group (younger vs. older adults) as a between‐subjects factor and Condition (global vs. local) as a within‐subjects factor, as shown in Equation (15):

(15) Recurrencemetricvalue∼Group×Condition+(1|Subject)

A random intercept for each subject accounted for repeated measures. Fixed effects were evaluated using F‐tests with Satterthwaite‐approximated degrees of freedom. For each metric, we extracted *p*‐values for (i) the main effect of Group, (ii) the main effect of Condition, and (iii) the Group × Condition interaction. To control for multiple comparisons, all *p*‐values across metrics and effects (Group, Condition, Interaction) were pooled and corrected using the Benjamini–Hochberg False Discovery Rate (FDR) procedure [105]. Reported *p*‐values therefore reflect correction across the full family of tests. For visualization, violin plots were generated for each metric, showing the distribution of values for the four combinations of Group and Condition. Means and variability were displayed within each violin plot to aid interpretation.

### 5.10 Spatial Gradient Embedding and Clustering Analysis of Network Topographies

To examine the spatial organization of the dominant brain networks identified via the BROAD‐NESS framework, we performed a spatial gradient embedding and clustering analysis on a voxel‐wise activation map. This analysis was conducted on the data averaged across participants to obtain a robust, group‐level representation of network topographies. We focused on the first three principal components (PC #1 – PC #3), as they represented the most important brain networks given calculations of effective dimensionality. For each component, we thresholded the voxel‐wise spatial weights by setting values between mean ± 1 standard deviation to zero. This allowed us to retain only the most strongly contributing voxels. These thresholded values were then used to define a 3D embedding space, where the position of each voxel was determined by its weight on PC #1 (x‐axis), PC #2 (y‐axis), and PC #3 (z‐axis). This provided a gradient‐like spatial representation, capturing how individual voxels contributed to the three brain networks. To identify spatially coherent voxel clusters within this 3D space, we applied k‐means clustering [106] to the embedded voxel coordinates. Prior to clustering, the data were z‐scored to normalize both axes and ensure equal weighting of the three components. For each value of *k* (ranging from 2 to 40) [107], we computed the k‐means clustering in 100 repetitions with different random initializations to minimize the effect of convergence to local minima [106]. To determine the optimal number of clusters, we computed the silhouette coefficient [108] for each clustering solution. This metric assesses how well‐separated the resulting clusters are by comparing the average distance between points within the same cluster to the average distance between points in neighboring clusters. Higher silhouette values indicate better‐defined, more distinct clusters. However, due to the stochastic nature of k‐means initialization, the silhouette scores can vary slightly across runs. To account for this variability and ensure robust estimation of the optimal number of clusters, we computed silhouette scores 5,000 times. The final clustering solution was selected based on the number of clusters that was most frequently associated with the highest silhouette score across these repetitions. This method provided a robust and data‐driven way to quantify the spatial gradients underlying the network topographies derived by the BROAD‐NESS method. Crucially, it enabled the identification of consistent and interpretable voxel clusters, including those that predominantly contributed to one network, those shared between two or three networks, and those with negative or minimal contributions. This approach offers a principled means to uncover the fine‐grained spatial architecture of large‐scale functional brain networks.

### 5.11 Investigating BROAD‐NESS Networks in Group and Condition Separated Data

To enhance the robustness of our work and provide greater insight into the subtle differences associated with the computation of PCA on neural data, we examined a crucial aspect relevant to experimental settings. Specifically, when multiple participant groups and experimental conditions are involved, it is essential to determine whether it is more beneficial to compute PCA on group‐ and condition‐separated data or on data aggregated across groups and conditions.

To address this, we computed both approaches and compared the results. Upon applying PCA to data averaged across conditions and groups in the main analysis, we computed PCA independently for each group in each condition. This allowed us to compare the brain networks and resulting time series computed using these two approaches and assess potential differences. In both approaches, the time series of brain networks were reconstructed independently for each participant using the weights of the activation matrix obtained through each method.

Specifically in the case of group‐ and condition‐separated data, the original MEG data were organized as a 4D array with dimensions *sources × time × condition × participants* and were split into four subsets corresponding to younger‐local, younger‐global, older‐local, and older‐global data. This approach allowed us to evaluate whether the dimensional structure, temporal expression, and spatial organization of BROAD‐NESS networks were preserved when network estimation was performed on more constrained group‐ and condition‐specific datasets.

Participants were assigned to younger and older groups based on predefined aging criteria, and data were indexed to create four 4D matrices of size *sources × time × 1 × participants*, providing one matrix per group–condition combination. These matrices were then used as input to the BROAD‐NESS pipeline, which computes group‐level network estimates while retaining participant‐specific time series for subsequent statistical analyses.

Within each group–condition dataset, PCA was applied to the group‐averaged MEG data to derive principal components capturing dominant patterns of covariation across brain sources over time. BROAD‐NESS returns (i) an eigenspectrum depicting the variance explained by each component and (ii) the corresponding brain network time series for each participant. For each decomposition, we inspected the full eigenspectrum and quantified effective dimensionality from the eigenvalue distribution, but focused interpretation on the first three components, which consistently accounted for the largest portion of explained variance across datasets.

For each retained component, we examined (i) the associated temporal profile of the network time series and (ii) the spatial activation pattern across brain sources. Network time series were extracted and used to characterize event‐related modulation. Spatial activation patterns were visualized by projecting component weights onto an 8 mm MNI152 template using the provided MNI voxel coordinates, yielding network topographies for each group‐condition‐specific decomposition. This further allowed for a qualitative comparison of spatial network structures across groups and conditions.

To evaluate differences between groups and conditions in the temporal expression of the BROAD‐NESS networks, we subjected the time courses of the first three components to timepoint‐wise 2 × 2 analyses (reflecting groups and conditions) using cluster‐based permutation testing. For each principal component, we contrasted group means (averaged over conditions), condition differences (global–local), and their interaction across the 0–800 ms time window, forming clusters of contiguous time points exceeding a cluster‐forming threshold. Family‐wise error was controlled via permutation‐derived maximum cluster mass distributions.

The rationale of this analysis was: (i) To investigate whether effects observed in the main BROAD‐NESS analysis were robust when network estimation was recomputed on group‐ and condition‐specific data. (ii) To investigate potential nuanced differences in time series expressions and activation patterns when applying BROAD‐NESS at more specific partitions of the data.

### 5.12 Brain Network Modularity Analysis

To characterize the modular organization of large‐scale brain network activity, we estimated temporally coherent large‐scale network components using principal component analysis (PCA) on the covariance structure of data from each individual. For each participant and each condition, we obtained a sorted eigenspectrum depicting the proportion of variance explained by each principal component. This procedure was applied independently for all participants, producing one eigenspectrum per subject and condition.

To quantify the degree to which activity patterns were concentrated vs. broadly distributed across components, we derived an estimate of effective dimensionality [51] from the eigenspectrum.

Effective dimensionality was quantified using the participation ratio of the principal component eigenvalue spectrum. Following principal component analysis (PCA) of the BROAD‐NESS brain network variance matrix, the vector of component variances λ_*i*_ was extracted. Effective dimensionality (ED) was computed as:

(16) $$ED=\frac{{\left(\sum_{i=1}^{k}\lambda_{i}\right)}^{2}}{\sum_{i=1}^{k}{\lambda_{i}}^{2}}$$

This measure reflects the number of components that effectively contribute to the variance structure, independent of the nominal dimensionality. Eigenvalues were constrained to be non‐negative prior to computation. The measure is bounded between 1 and the total number of components.

Conceptually, higher ED values indicate that the total variance is distributed across a larger set of components, whereas lower values indicate that the variance is captured by a smaller, more compact network structure. ED was calculated for each participant in each condition. We visualized the distribution of ED values using scatter plots at the single‐subject level and violin plots at the group level. Group differences were tested using a 2 × 2 mixed ANOVA with Group (younger, older) as a between‐subject factor and Condition (local, global) as a within‐subject factor. *p*‐values were Bonferroni‐corrected across the three main ANOVA effects.

To relate ED to the underlying eigenspectra, we also computed cumulative variance explained curves for each condition and group. For each participant, cumulative variance was calculated for the first 30 principal components. Group means and standard errors were derived separately for younger and older adults. Vertical reference lines were plotted at the group level to illustrate how mean ED values corresponded to plotted curves indicating cumulative variance explained across principal components.

## Author Contributions

L.B., M.R. and M.H.A. conceived the hypotheses. L.B., G.F.R. and D.R.Q.M. designed the study. L.B., M.L.K. and P.V. recruited the resources for performing data collection and analysis. D.R.Q.M. designed the experimental paradigm used in the study. G.F.R., M.K. and L.B. collected the data. M.H.A. and L.B. performed pre‐processing, statistical analysis and decomposition of the neural signal. L.B. and M.H.A. updated the code for the BROAD‐NESS toolbox. M.L.K., P.V., H.R.S., K.M.L. and M.R. provided essential help to interpret and frame the results within the neuroscientific and analytical literature. M.H.A. wrote the first draft of the manuscript, which was primarily integrated by L.B. and M.R. The figures were prepared by M.H.A., L.B. and M.R. All the authors contributed to and approved the final version of the manuscript.

## Conflicts of Interest

Hartwig R. Siebner has received honoraria as editor (Neuroimage Clinical) from Elsevier Publishers, Amsterdam, The Netherlands. He has received royalties as book editor from Springer Publishers, Stuttgart, Germany, Oxford University Press, Oxford, UK, and from Gyldendal Publishers, Copenhagen, Denmark. The other authors declare no competing interests.

## Code Availability Statement

The BROAD‐NESS toolbox is available at the following link and is required to run the data analysis pipeline: https://github.com/leonardob92/BROADNESS_MEG_AuditoryRecognition/tree/main/BROADNESS_Toolbox The main data analysis pipeline used in this study is available at the following link: https://github.com/MathiasHoueAndersen/Predictive‐Processing‐In‐Aging In‐house‐built code and functions used for the pre‐processing of MEG data in this study are part of the LBPD repository which is available at the following link: https://github.com/leonardob92/BROADNESS_Aging_MMN_AdvancedScience.git.

## Supporting information




**Supporting File 1**: advs77857‐sup‐0001‐SuppMat.docx.

**Supporting File 2**: advs77857‐sup‐0001‐Tables.zip.

## Data Availability

The pre‐processed neuroimaging data generated in this study have been deposited in the Zenodo database and are publicly available: https://doi.org/10.5281/zenodo.18231641 [109]. The code used to present the stimuli of the experimental paradigm used in this study has been deposited on GitHub and is publicly available: https://github.com/MathiasHoueAndersen/Hierarchical‐Predictive‐Processing/blob/main/Experimental_Paradigm.py
