# Supplementary material for: Brain Network Dynamics of Local and Global Predictive Processing in Aging
Source: Adv Sci (Weinh). 2026 Sep 27:e77857. Online ahead of print. doi: 10.1002/advs.77857 (PMC13616317; doi:10.1002/advs.77857)
Supplement: Supplementary file 1 [file ADVS-9999-e77857-s002.docx]

*Supplementary Information for*

**Brain Network Dynamics of Local and Global Predictive Processing in Aging**

*Mathias Houe Andersen _1, 2, 3_*_,_ Gemma Fernández-Rubio _1,_ David R. Quiroga-Martinez _1,4,_*

*Mattia Rosso _1,5,_ Mathias Klarlund _1,_* *Kit Melissa Larsen _2,_ Hartwig Roman Siebner _2,3,6,_*

*Morten L. Kringelbach _1,7,8,_ Peter Vuust _1,_ Leonardo Bonetti _1,7,8_**

^1^ Center for Music in the Brain, Department of Clinical Medicine, Aarhus University & The Royal Academy of Music, Aarhus/Aalborg, Denmark

^2^ Danish Research Centre for Magnetic Resonance, Department of Radiology and Nuclear Medicine, Copenhagen University Hospital – Amager and Hvidovre, Hvidovre, Denmark

^3^ Faculty of Health and Medical Sciences, University of Copenhagen, Copenhagen, Denmark

^4^ Department of Psychology, University of Copenhagen, Copenhagen, Denmark

^5^ IPEM Institute for Systematic Musicology, Ghent University, Ghent, Belgium

^6^ Department of Neurology, Copenhagen University Hospital Bispebjerg and Frederiksberg, Copenhagen, Denmark

^7^ Department of Psychiatry, University of Oxford, Oxford, United Kingdom

^8^ Centre for Eudaimonia and Human Flourishing, Linacre College, University of Oxford, Oxford, United Kingdom

*Corresponding authors: mathiasha@drcmr.dk (M.H.A.), [leonardo.bonetti@psych.ox.ac.uk](mailto:leonardo.bonetti@psych.ox.ac.uk) (L.B.)

ORCID:

https://orcid.org/0009-0003-2513-5251

https://orcid.org/0000-0001-9983-3819

**Supplementary figures**

The same supplementary figures are available in high resolution at the following link:

<https://doi.org/10.5281/zenodo.21278203>

**Figure S1 | The auditory local-global paradigm.**

Each trial consisted of patterns of five tones. The duration of each tone was 80 ms separated by 20 ms of silence yielding a stimulus onset asynchrony of 100 ms. Each five-tone pattern was followed by a 500 ms inter-trial interval. Tone patterns could be either five identical tones (local standards) or sequences in which the fifth tone differed in pitch (local deviants). Complementing this experimental fifth tone that induces local sensory violations, a target tone requiring a button press was also rarely presented to ensure continued attention towards the paradigm. Blocks manipulated prediction violations at two hierarchical levels. In congruent blocks, local standards occurred most frequently (70%), local experimental deviants occurred less often (23.75%), and target tones appeared rarely (6.25%). In incongruent blocks, the probabilities of local standards and local deviants were switched, creating a global violation in pattern frequency, while target probability remained constant. Participants completed two congruent and two incongruent blocks in randomized order. Three pitch levels were used (standard tones: 440 Hz; deviating tones: 4 semitones below standards, ~349 Hz; target tones: 10 semitones above standards, ~784 Hz). This design elicits local prediction errors at the tone level and global prediction errors at the pattern level, while maintaining attention through infrequent targets.

**Figure S2 | Event-related field components identification.**

This figure illustrates the identification of canonical event-related field (ERF) components from group-mean brain network time series derived from principal component (PC) decomposition. Mean activation time courses (μ ± SE) are shown separately for younger and older participants under local and global conditions, with PC #1–3 distinguished by line style. ERF components were detected by extracting the most positive or negative peak within predefined latency windows: for the local condition, P50 (50 – 100 ms, positive), MMN (100 – 200 ms, negative), P300 (150 – 300 ms, positive), and RON (250 – 400 ms, negative); for the global condition, MMN (100 – 200 ms, negative), P300 (150 – 250 ms, positive), and RON (250 – 400 ms, negative). Peak latencies are marked with symbols on the corresponding time series. Components that were not reliably identifiable were excluded a priori (e.g., RON in PC #2 for the local condition, P300 in PC #2 for the global condition, and P50 in the global condition), and global RON was only quantified for PC #1. These peak estimates form the basis for the latency, amplitude, and temporal order summary reported in Table S4.

**Figure S3.1 | Phase space trajectories in two dimensions.**

Phase space trajectories averaged across participants for each experimental condition and age group, obtained by embedding the time series of the three primary BROAD-NESS networks into a two-dimensional state space in all three combinations possible. Each dot represents the state of the brain at a given time point, colour-coded by time (in seconds). Across the 3 different comparisons (PC #1 vs. PC #2, PC #2 vs. PC #3 and PC #1 vs. PC #3), in the local condition younger adults show a more sharply defined time specific coactivation and deactivation of singular networks compared to slower and less distinct transitions observed among older adults. Both groups display a more concentrated time specific activation and deactivation of individual networks in the local compared to the global condition.

**Figure S3.2 | Deviations in phase space trajectory.**

Plots showing deviations in each dimension (PC #1, PC # 2, PC #3) of the three-dimensional phase space trajectories between age groups in each condition (left, center) and differences between the conditions across age groups (right). These plots can be used qualitatively assess deviations in each dimension of the three-dimensional phase space trajectory.

**Figure S4 | Recurrence plots without threshold.**

Recurrence plots computed from the three-dimensional phase space trajectories in Figure 3b. The matrices are averaged across participants, depicting pairwise Euclidean distances between time points. Blue indicates larger distances (i.e., less similar brain states), while yellow indicate greater similarity and temporal recurrence. Across both conditions, younger adults show less recurrence of the three brain networks across time compared to older adults indicating more exploratory and less static temporal dynamics regarding the reutilisation of brain networks.

**Figure S5 | Voxel-wise clustering solutions for spatial gradients embedding of BROAD-NESS-derived brain networks (k = 2 to 40).**

Each subplot shows a 3D embedding of brain voxels based on their contribution to the three principal BROAD-NESS networks, with colour-coded clusters derived from unsupervised k-means clustering for values of k ranging from 1 to 40. The embedding reflects how individual voxels co-participate across networks, revealing patterns of overlap, selectivity, or opposition. The full panel allows visual comparison across clustering solutions to identify consistent subdivisions the brain voxels across multiple runs of clustering analysis. The optimal number of clusters, determined through silhouette scores and repetition stability (corresponding to k = 16, is reported both here and in higher detail in the main text.

**Figure S6 | Evaluation of cluster solutions.**

**a**, Histogram of the frequency with which each *k* was selected as silhouette-optimal across 5,000 repetitions for each tested cluster solution (*k* = 2 to 40) of the spatial gradients embedding of BROAD-NESS-derived brain networks. The peak at *k* = 16 confirms the optimal number of spatial clusters, based on maximum silhouette coefficient consistency. **b**, The plot shows the within-cluster sum of distances (SUM; k-means objective) as a function of the number of clusters *k* (here, *k* = 2 to 40), computed from voxel embeddings in principal-component weight space after thresholding and z-scoring. For each *k*, k-means clustering was run with 100 replicates with different random initializations, and the best solution with the lowest within-cluster dispersion across replicates was retained. SUM decreases monotonically as *k* increases since additional clusters allow a progressively finer partitioning of the embedding space.

**Figure S7 | Sensor-level analysis**

The figure depicts ERF responses at sensor level in younger and older participants. Analyses were performed on mismatch responses obtained from the difference wave between standard and deviant trials. Difference waves were extracted from eight predefined sensors over bilateral frontotemporal regions (MEG0131, MEG0121, MEG0231, MEG0241, MEG1331, MEG1341, MEG1611, and MEG1621). To combine pairs of sensors, the polarity of signals from one sensor set (MEG0231/0241 and MEG1611/1621) was flipped prior to averaging. Signals were first averaged across sensors within each hemisphere, then averaged across hemispheres to obtain a single sensor-level ERF time series for each participant in each condition. For each participant, ERFs were obtained separately for the Global and Local conditions. Group-average ERFs were then computed for each combination of age group and condition, resulting in four-time courses: Younger–Local, Older–Local, Younger–Global, and Older–Global. Shaded ribbons surrounding each trace represent the standard error of the mean across participants. The analysis window extended from −0.1 to 0.8 s relative to the onset of the fifth tone in the local–global sequence, with time 0 s marking presentation of the fifth tone. Statistical inference was performed using cluster-based permutation testing across time with the same approach as in the main analysis on source-reconstructed data. Three effects were evaluated: a group main effect (younger versus older participants), a condition main effect (Global versus Local conditions), and a Group × Condition interaction. For the group main effect, condition-averaged responses were compared between groups using label permutation. For the condition main effect, within-subject Global–Local difference waves were tested using random sign-flipping across participants. For the interaction effect, Global–Local difference waves were compared between groups using between-group permutation testing. In all cases, cluster-forming thresholds were derived from the empirical permutation distributions (cluster-forming α = 0.05), and significance was assessed using the maximum cluster-mass statistic with family-wise error rate control at α = 0.05 based on 5,000 permutations. Grey shaded regions indicate time intervals in which significant clusters were detected for at least one of the tested effects. Coloured horizontal bars at the bottom of the plot indicate the temporal extent of significant clusters for each specific effect: green denotes the group main effect, purple denotes the condition main effect, and orange denotes the interaction effect. These intervals correspond to cluster-corrected significant effects identified by the permutation analyses. Significant condition effects were observed over extended portions of the post-stimulus interval, while group and interaction effects emerged during more restricted temporal windows. Together, these results demonstrate the robustness of the results obtained from the main analysis, where BROAD-NESS was applied to source-reconstructed data, as both age group and hierarchical auditory context influence the neural responses recorded at the sensor-level in a very similar manner. For the results of the statistical test used to examine group, condition and interaction differences see Table S13.2.

**Figure S8 | Behavioural data results.**

The figure shows behavioural performance during the auditory local–global paradigm for younger and older adults. Individual participant values are displayed as dots, while boxplots summarize the distribution within each age group. **a**, The number of correctly detected target tones (hits), where 40 is the maximum. Here, both younger and older adults achieved near-ceiling performance, with most participants correctly detecting almost all target tones presented during the task. A small number of participants in both groups showed lower hit counts, though overall accuracy remained high across age groups. **b**, The number of missed target tones (misses), where 40 is the maximum. Here, misses were generally infrequent for both younger and older adults, indicating that participants successfully maintained attention throughout the paradigm. Nevertheless, a small subset of older adults exhibited substantially higher miss counts than the majority of participants. **c**, The number of button presses made in the absence of a target tone (false alarms). False alarms were defined a button press within a two second time span following each of the 40 target tones presented in the paradigm. This time span covered the trial of the target tone and the subsequent trial, which was specifically programmed to not play another target tone. A modest number of false alarms did occur for most participants (${Median}_{younger}$ = 4, ${Median}_{older}$ = 8), while both age groups contained some individuals with markedly elevated false-alarm rates. **d**, The mean reaction times for correctly detected targets. Here, the median and overall distribution of the older adult responses were shifted toward slower levels. Reaction times among older adults also appeared more variable, whereas younger adults showed faster and more tightly clustered responses. The behavioural measures were included to assess task engagement and performance during the experiment. Based on a threshold of deviating +/- 3 SD from the mean in either hits or false alarms, 7 participants were classified as outliers. Across the whole sample, 3 participants were excluded due to their number of hits, while 4 participants were excluded due to their number of false alarms. These participants were excluded in a follow-up robustness analysis of the main results, to ensure that these results were not due to a potential lack of engagement in the task. Across the whole sample of participants, Mann–Whitney U tests were conducted to compare behavioural performance between younger and older adults. This non-parametric test was utilised as at least one age group showed a non-normal distribution in all three tested behavioural measures (hits, false alarms and reaction time). Significant age group differences were observed for the number of hits (younger: M = 38.84, SD = 6.57, median = 40.00, n = 37; older: M = 37.03, SD = 9.13, median = 40.00, n = 39; U = 905.50, p = .010, pFDR = .015) and false alarms (younger: M = 28.49, SD = 87.64, median = 4.00, n = 37; older: M = 37.54, SD = 80.22, median = 8.00, n = 39; U = 529.50, p = .044, pFDR = .044). This provided evidence, that older adults displayed significantly less hits and more false alarms compared to the younger adults. Older adults also showed significantly slower reaction times than younger adults (younger: M = 0.79, SD = 0.07, median = 0.78, n = 36; older: M = 0.83, SD = 0.07, median = 0.82, n = 37; U = 430.00, p = .009, pFDR = .015). Thus, significant age group differences were observed for hits, false alarms and reaction times, and all three effects remained significant after false discovery rate correction. Despite these age-related differences in false alarms and hits, overall performance indicated that most participants remained attentive and engaged with the task, with the modestly poorer performance observed in older adults being consistent with generally expected age-related differences in behavioural performance. One older participant missed behavioural data, while two older participants and one younger participant on average did not respond within the 2 second post-stimulus threshold chosen to define responses. Therefore, these participants did not contribute to these tests or to the graphical plotting of the results. See Table S13.1 to inspect all outputs of the statistical tests comparing these three measures between the two age groups. Significance levels: * p < .05, ** p < .01, *** p < .001; all asterisk-denoted p-values were significant after false discovery rate (FDR) correction.

**Figure S9 | Robustness analysis of main results excluding behavioural outliers**

The figure depicts the robustness analysis of the main results, after 7 participants were removed due to being outliers in task performance (see Figure S8). The figure illustrates time series and spatial activation patterns of the three brain networks that explained the highest variance (51.49 %, 24.27 % and 15.65 % in the robustness analysis; 51.78 %, 24.57 % and 15.12 %, respectively in the main analysis). These networks were estimated using PCA within the BROAD-NESS framework, computed on the data averaged across conditions and participants. **a**, Independent time series for each participant, brain network, and experimental condition were generated using PCA-derived weights from the averaged data. The individual time series were then averaged across participants, as shown in the plots. Shaded areas represent standard errors of the mean. The brain templates illustrate the spatial extent of the networks, with yellow voxels contributing the most and light blue voxels contributing the least to the time series. Only voxels with values exceeding the mean by more than one standard deviation in absolute terms are depicted. Significant group, condition and interaction effects are indicated by green, purple and yellow lines. Time = 0 s. indicates the onset of the fifth tone in the local-global paradigm. **b**, The eigenspectrum for the first 10 principal components and effective dimensionality (ED = 2.860 in the robustness analysis; ED = 2.835 in the main analysis), indicates the relevance of the first three principal components (dashed line). The overall finding from this robustness analysis was that excluding behavioural outliers only marginally altered explained variance, effective dimensionality and spatial activation patterns. In contrast, statistical testing in the robustness analysis showed fewer group effects due to a loss of power by removing behavioural outliers. For the results of the statistical test used to examine group, condition and interaction differences see Table S12.

**Figure S10.1 | Half-split analysis - Eigenspectra**

To assess the stability of the latent dimensional structure identified by BROAD-NESS, eigenspectra were calculated separately for the first and second halves of trials. The figure shows the percentage of variance explained by the first ten principal components in each half-split dataset. Effective dimensionality was estimated from the full variance spectrum and is indicated by the dashed vertical line. The eigenspectra were highly similar across both trial halves, with the majority of variance captured by the first three to four components and a steep decline thereafter. Effective dimensionality estimates were comparable between splits (ED = 3.395 and ED = 2.933), indicating a stable low-dimensional network structure. Together, these results demonstrate that the dimensional organization identified by the BROAD-NESS decomposition is highly reproducible across independent subsets of trials.

**Figure S10.2 | Half-split analysis**

To evaluate the stability of the BROAD-NESS decomposition, all analyses were repeated independently on two non-overlapping halves of the trial data. The temporal activation profiles of the first three brain networks are shown separately for the first and second halves of trials. Mean network activations are displayed for younger and older participants under local and global deviant conditions, with shaded regions indicating the standard error of the mean. Statistical effects were assessed using cluster-based permutation tests (5,000 permutations) examining main effects of group, condition, and their interaction. Significant clusters are indicated by coloured bars beneath the waveforms and corresponding grey shaded regions. Across all three networks, highly similar temporal activation profiles and spatial topographies were observed in both trial halves. The timing and overall pattern of significant condition, group, and interaction effects were largely preserved across splits, indicating that the identified brain networks and their associated statistical effects were robust and reproducible. For the results of the statistical test used to examine group, condition and interaction differences see Table S14.1.

**Supplementary Tables**

The supplementary tables are available at the following link:

<https://doi.org/10.5281/zenodo.21278444>

**Table S1 | MNI coordinates of brain voxels from the three main brain networks.**

This table reports the results of the voxels corresponding to the three main brain networks derived from BROAD-NESS in the main analysis. For each voxel, the table provides its progressive index and MNI coordinates (X, Y, Z), along with its corresponding values (spatial activation pattern) on PC #1, PC #2 and PC #3.

**Table S2 | Labels of brain areas within the three main brain networks.**

This table summarizes voxel-level contributions from each brain network after mapping MNI-space coordinates to anatomical regions defined by the AAL 3.1 atlas^[1]^. Voxels were first separated into positive and negative contributions based on the sign of the network loading. Each voxel was assigned an atlas label using exact voxel overlap when possible; voxels falling in background or unlabelled regions were iteratively associated with the nearest non-zero atlas label within a predefined voxel radius. Counts are reported per brain area and stratified by the radius at which a valid label was obtained (exact match, 1-voxel shell, 2-voxel shell, 3-voxel shell), with unresolved voxels tracked separately. Distances are defined in atlas voxel space using a Chebyshev neighbourhood metric, where distance is determined by the largest difference in coordinates along any axis. Additional summary tables report the overall distribution of brain area labelling across voxels.

**Table S3 | Statistical analysis of the timeseries from the three main brain networks.**

This table presents the results of statistical analyses conducted independently for the three brain networks that explained the highest variance. Time series of these principal components were analysed in a mixed design with the between-subject factor Group (younger vs. older adults) and the within-subject factor Condition (local vs. global) in the 0 – 800 ms post-stimulus interval. Specifically, we derived three contrast time series per network: (i) a Group main effect (younger minus older, averaging across local and global), (ii) a Condition main effect (global minus local, pooling across age groups), and (iii) a Group × Condition interaction (the difference of the global–local contrast between younger and older adults). For each contrast and each time point, we applied a two-sided cluster-based permutation test over time spanning 5,000 permutations.

**Table S4.1 | Latency, order and amplitude of event-related field components.**

This table summarizes the peak latency, peak amplitude, and temporal order of event-related field components derived from group-mean time series, reported separately for younger and older participants and for the local and global condition. Event-related field components were quantified from the mean signal (μ) of each principal component, averaged across participants. Component amplitudes were defined within predefined latency windows as either the most positive or negative value depending on the component polarity. For the local condition, P50 (50–100 ms, positive), MMN (100–200 ms, negative), P300 (150–300 ms, positive), and RON (250–400 ms, negative) were analysed. For the global condition, MMN (100–200 ms, negative) and P300 (150–250 ms, positive) were considered. Some event-related field components were not easily identified in the time series and were therefore excluded a priori. This included the RON response for PC #2 in the local condition, and P300 for PC #2 in the global condition. Similarly, in the global condition P50 was not clearly identified in any PC, while the RON response was only identified in PC #1.

**Table S4.2 | Participant-level event-related field component latencies and amplitudes.**

This table summarizes participant-level latency and amplitude characteristics of ERF components identified within predefined temporal windows for each principal component within each condition, and age group. For each component, the number of valid observations, missing-data rate, mean and standard deviation of latency, median latency, interquartile range, and mean absolute amplitude are reported. Values are presented separately for younger and older participants and for the local and global condition.

**Table S4.3 | Pairwise comparisons of principal component latency order.**

This table reports pairwise Wilcoxon signed-rank tests comparing ERF component latencies between principal components (PC #1, PC #2, PC #3). Analyses were performed separately for each condition and component using participant-level latency estimates. Mean and median latency differences are reported together with uncorrected and false-discovery-rate (FDR) corrected p-values to assess whether specific brain networks were activated earlier or later than others for each ERF component.

**Table S4.4 | Temporal ordering of principal component responses.**

This table summarizes the temporal sequence of ERF components across principal components. For each condition and component, mean, standard deviation, and median latencies are reported for PC #1, PC #2 and PC #3, together with the observed latency order. Statistical evidence for differences in latency across principal components was assessed using Friedman tests, providing an overall test of whether the timing of ERF components differed significantly across brain networks.

**Table S5.1 | Average 3D phase space trajectory values.**

This table contains time-resolved, group-averaged phase-space coordinates derived from recurrence quantification analysis (RQA) corresponding to both age groups (young, older) and conditions (local, global). Rows represent the time vector followed by the first three principal component (PC) coordinates defining the reconstructed phase space. Columns correspond to successive time points. Values are provided in arbitrary units reflecting PCA-derived component scores. The data enable exact numerical inspection of the reported phase-space plots and allow independent analysis of trajectory geometry.

**Table S5.2 | Phase space statistics – age-related permutation test results, local condition.**

This table provides complete statistical details for the age group comparison of the phase-space trajectories in the local condition. The table contains analysis settings, time-point-wise statistics, observed cluster information, permutation-derived null distributions, mean phase-space trajectories for younger and older adults, and individual participant coordinates in the three-dimensional phase space. Time-resolved statistics include observed Euclidean distances between group mean trajectories, cluster-forming thresholds, uncorrected p-values, cluster assignments, cluster-corrected p-values, and significance status.

**Table S5.3 | Phase space statistics – age-related permutation test results, global condition.**

This table provides complete statistical details for the age group comparison of the phase-space trajectories in the global condition. The table contains analysis settings, time-point-wise statistics, observed cluster information, permutation-derived null distributions, mean phase-space trajectories for younger and older adults, and individual participant coordinates in the three-dimensional phase space. Time-resolved statistics include observed Euclidean distances between group mean trajectories, cluster-forming thresholds, uncorrected p-values, cluster assignments, cluster-corrected p-values, and significance status.

**Table S5.4 | Phase space statistics – clusters of age group differences.**

This table reports the results of cluster-based permutation analyses testing age group differences in the three-dimensional phase-space trajectories. Analyses were performed separately for the local and global condition. At each time point, the Euclidean distance between the younger and older adults’ mean trajectories in the three-dimensional phase space (PC #1, PC #2, PC #3) was calculated. Statistical significance was assessed using cluster-based permutation testing with 5,000 permutations and correction for multiple comparisons across time. For each observed cluster, the table reports the cluster onset and offset times, duration, cluster mass, cluster-level p-value, and significance after cluster correction.

**Table S5.5 | Phase space statistics – condition-wise permutation test results across all participants.**

This table provides complete statistical details for the condition-wise comparison of local and global phase-space trajectories across all participants. The table contains analysis settings, time-point-wise statistics, observed cluster information, permutation-derived null distributions, mean local and global trajectories, and participant-level phase-space coordinates. Statistical testing was based on the Euclidean distance between Local and Global mean trajectories in three-dimensional phase space (PC #1, PC #2, PC #3). Significance was evaluated using paired cluster-based permutation testing with 5,000 permutations, preserving participant pairing through within-subject label exchange. Time-resolved statistics include observed distances, cluster-forming thresholds, uncorrected p-values, cluster assignments, cluster-corrected p-values, and significance status, enabling complete reproduction of the reported local-versus-global phase-space analyses.

**Table S5.6 | Phase space statistics – clusters of condition differences.**

This table reports the results of cluster-based permutation analyses testing differences between local and global three-dimensional phase-space trajectories across all participants. At each time point, the Euclidean distance between the mean local and global trajectories in the three-dimensional phase space (PC #1, PC #2, PC #3) was calculated. Statistical significance was assessed using paired cluster-based permutation testing with 5,000 permutations, where local and global labels were randomly exchanged within participants while preserving participant pairing. For each observed cluster, the table reports the cluster onset and offset times, duration, cluster mass, cluster-level p-value, and significance after cluster correction.

**Table S6 | MNI coordinates of brain voxels assigned to each cluster in the spatial gradient embedding analysis.**

This table reports the results of the voxel-wise clustering analysis based on the spatial gradient embedding of the three principal BROAD-NESS networks. Voxels were clustered according to their contributions to Network #1, Network #2 and Network #3 (represented by PCA components PC #1, PC # and PC #3, respectively), using an unsupervised k-means clustering approach. The optimal solution identified 16 clusters, each shown in a separate sheet. For each voxel, the table provides its progressive index and MNI coordinates (X, Y, Z), along with its corresponding values (spatial activation pattern) on PC #1, PC #2 and PC #3.

**Table S7 | Labels of brain areas for each cluster in the spatial gradient embedding analysis.**

This table summarizes voxel-level contributions from each brain network after mapping MNI-space coordinates to anatomical regions defined by the AAL 3.1 atlas^i^. Each voxel was assigned an atlas label using exact voxel overlap when possible; voxels falling in background or unlabelled regions were iteratively associated with the nearest non-zero atlas label within a predefined voxel radius. Counts are reported per brain area and stratified by the radius at which a valid label was obtained (exact match, 1-voxel shell, 2-voxel shell, 3-voxel shell), with unresolved voxels tracked separately. Distances are defined in atlas voxel space using a Chebyshev neighbourhood metric, where distance is determined by the largest difference in coordinates along any axis. Additional summary tables report the overall distribution of brain area labelling across voxels.

**Table S8 | Minimum and maximum values of brain network loadings in each cluster in the spatial gradient embedding analysis.**

This table reports the results of the voxel-wise clustering analysis based on the spatial gradient embedding of the three principal BROAD-NESS networks. Voxels were clustered according to their contributions to Brain Network #1, Brain Network #2 and Brain Network #3 (represented by PCA components PC #1, PC # and PC #3, respectively), using an unsupervised k-means clustering approach. The optimal solution identified 16 clusters. The table shows the minimum and maximum values of the loadings on each network within each of the 16 clusters. Xmin and Xmax refer to PC #1 loading boundaries, Ymin and Ymax refer to PC #2 loading boundaries, while Zmin and Zmax refer to PC #3 loading boundaries.

**Table S9 | Statistical analysis of the timeseries from brain networks when BROAD-NESS is applied to group and condition separated data.**

This table presents the results of statistical analyses conducted independently for the three brain networks within each group-condition-combination when BROAD-NESS was applied to group and condition segregated data. Time series of these principal components were analysed in a mixed design with the between-subject factor Group (younger vs. older adults) and the within-subject factor Condition (local vs. global) in the 0 – 800 ms post-stimulus interval. Specifically, we derived three contrast time series per network: (i) a Group main effect (younger minus older, averaging across local and global), (ii) a Condition main effect (global minus local, pooling across age groups), and (iii) a Group × Condition interaction (the difference of the global–local contrast between younger and older adults). For each contrast and each time point, we applied a two-sided cluster-based permutation test over time spanning 5,000 permutations.

**Table S10 | MNI coordinates of brain voxels from the three main brain networks when BROAD-NESS is applied to group and condition separated data.**

This table reports the results of the voxels corresponding to the three main brain networks when BROAD-NESS is applied to group and condition separated data. For each voxel, the table provides its progressive index and MNI coordinates (X, Y, Z), along with its corresponding values (spatial activation pattern) on PC #1, PC #2 and PC #3.

**Tables S11.1, S11.2, S11.3 & S11.4 | Labels of brain areas from brain networks when BROAD-NESS is applied to group and condition separated data.**

These four tables summarize voxel-level contributions from each brain network after mapping MNI-space coordinates to anatomical regions defined by the AAL 3.1 atlas. There is one table for each group and condition separated dataset. Voxels were first separated into positive and negative contributions based on the sign of the network loading. Each voxel was assigned an atlas label using exact voxel overlap when possible; voxels falling in background or unlabelled regions were iteratively associated with the nearest non-zero atlas label within a predefined voxel radius. Counts are reported per brain area and stratified by the radius at which a valid label was obtained (exact match, 1-voxel shell, 2-voxel shell, 3-voxel shell), with unresolved voxels tracked separately. Distances are defined in atlas voxel space using a Chebyshev neighbourhood metric, where distance is determined by the largest difference in coordinates along any axis. Additional summary tables report the overall distribution of brain area labelling across voxels.

**Table S12 | Statistical analysis of the timeseries from sensor-level data.**

This table presents the results of statistical analyses conducted on the sensor-level data within each group-condition-combination. The time series were composed from averages from 8 central sensors (see Figure S7 for details). These time series were analysed in a mixed design with the between-subject factor Group (younger vs. older adults) and the within-subject factor Condition (local vs. global) in the 0 – 800 ms post-stimulus interval. Specifically, we derived three contrast time series: (i) a Group main effect (younger minus older, averaging across local and global), (ii) a Condition main effect (global minus local, pooling across age groups), and (iii) a Group × Condition interaction (the difference of the global–local contrast between younger and older adults). For each contrast and each time point, we applied a two-sided cluster-based permutation test over time spanning 5,000 permutations.

**Table S13.1 | Statistical comparison of behavioural performance between younger and older adults.**

This table presents the results of independent-samples statistical comparisons of behavioural performance between younger and older adults. Three behavioural measures were analysed: mean reaction time (RT), number of hits, and number of false alarms. For each measure, normality was assessed separately in the younger and older groups using Lilliefors tests. If both groups met the normality assumption, Welch’s independent-samples *t*-test was used; if at least one group failed the normality assessment, a two-sided Mann–Whitney U test was performed. For all three behavioural measures, at least one group failed the normality assessment, and Mann–Whitney U tests were therefore used. The table reports the sample size (*n*), mean, standard deviation (SD), and median for each age group, together with the Lilliefors test statistic and *p*-value for each group, whether normality was rejected, the statistical test used, the corresponding test statistic, and the uncorrected *p*-value. To account for multiple comparisons across the three behavioural measures, *p*-values were additionally corrected using the false discovery rate (FDR) procedure, and statistical significance following FDR correction is indicated in the final column.

**Table S13.2 | Statistical analysis of the timeseries from brain networks when BROAD-NESS is applied to data where behavioural outliers are excluded.**

This table presents the results of statistical analyses conducted independently for the three brain networks within each group-condition-combination when BROAD-NESS was applied to data where behavioural outliers were excluded. Time series of these principal components were analysed in a mixed design with the between-subject factor Group (younger vs. older adults) and the within-subject factor Condition (local vs. global) in the 0 – 800 ms post-stimulus interval. Specifically, we derived three contrast time series per network: (i) a Group main effect (younger minus older, averaging across local and global), (ii) a Condition main effect (global minus local, pooling across age groups), and (iii) a Group × Condition interaction (the difference of the global–local contrast between younger and older adults). For each contrast and each time point, we applied a two-sided cluster-based permutation test over time spanning 5,000 permutations.

**Table S14.1 | Statistical analysis of the timeseries from brain networks when BROAD-NESS is applied to half-split data.**

This table presents the results of statistical analyses conducted independently for the three brain networks within each group-condition-combination when BROAD-NESS was applied to data, where trials in the first half of the session was separated from the last half of the session. See Figure S10.1 and S10.2 for details. Time series of these principal components were analysed in a mixed design with the between-subject factor Group (younger vs. older adults) and the within-subject factor Condition (local vs. global) in the 0 – 800 ms post-stimulus interval. Specifically, we derived three contrast time series per network: (i) a Group main effect (younger minus older, averaging across local and global), (ii) a Condition main effect (global minus local, pooling across age groups), and (iii) a Group × Condition interaction (the difference of the global–local contrast between younger and older adults). For each contrast and each time point, we applied a two-sided cluster-based permutation test over time spanning 5,000 permutations.

**Table S14.2 | Half-split permutation correlations**

To assess the stability of the BROAD-NESS decomposition, the data were repeatedly divided into two independent halves and the complete analysis was performed separately on each subset. Ten random split-half permutations were generated, together with one deterministic split comparing the first half of trials with the second half of trials. Reliability was quantified using Pearson correlations between corresponding components obtained from the two halves. Spatial Activation Pattern (SAP) values reflect correlations between the spatial maps of the three BROAD-NESS components (PC #1, PC #2, PC #3), whereas Time Series (TS) values reflect correlations between the corresponding network time courses for the Global and Local conditions. High correlation coefficients indicate that both the spatial organization and temporal dynamics of the identified networks were highly reproducible across independent subsets of the data. Negative correlations observed for some PC #3 estimates in the deterministic split likely reflect the arbitrary sign indeterminacy inherent to principal component decompositions rather than a difference in the underlying network structure.

**References**

1. Rolls, E. T., Huang, C. C., Lin, C. P., Feng, J. & Joliot, M. Automated anatomical labelling atlas 3. *NeuroImage* **206**, 116189 (2020). <https://doi.org/10.1016/j.neuroimage.2019.116189>
